# Discovery of a CNS penetrant small molecule *SMN2* splicing modulator with improved tolerability for spinal muscular atrophy

**DOI:** 10.1038/s41598-020-74346-9

**Published:** 2020-10-15

**Authors:** Shiori Ando, Shunya Suzuki, Shoichi Okubo, Kazuki Ohuchi, Kei Takahashi, Shinsuke Nakamura, Masamitsu Shimazawa, Koji Fuji, Hideaki Hara

**Affiliations:** 1 Molecular Pharmacology, Department of Biofunctional Evaluation, Gifu Pharmaceutical University, 1-25-4 Daigaku-nishi, Gifu, 501-1196 Japan; 2 Reborna Biosciences Inc., Kanagawa, 251-0012 Japan

**Keywords:** Drug discovery, Diseases

## Abstract

Spinal muscular atrophy (SMA) is a motor neuron disease, typically resulting from loss-of-function mutations in the survival motor neuron 1 (*SMN1*) gene. Nusinersen/SPINRAZA, a splice-switching oligonucleotide that modulates *SMN2* (a paralog of *SMN1*) splicing and consequently increases SMN protein levels, has a therapeutic effect for SMA. Previously reported small-molecule *SMN2* splicing modulators such as risdiplam/EVRYSDI and its analog SMN-C3 modulate not only the splicing of *SMN2* but also that of secondary splice targets, including forkhead box protein M1 (*FOXM1*). Through screening SMA patient-derived fibroblasts, a novel small molecule, designated TEC-1, was identified that selectively modulates *SMN2* splicing over three secondary splice targets. TEC-1 did not strongly affect the splicing of *FOXM1*, and unlike risdiplam, did not induce micronucleus formation. In addition, TEC-1 showed higher selectively on galactosylceramidase and huntingtin gene expression compared to previously reported compounds (e.g., SMN-C3) due to off-target effects on cryptic exon inclusion and nonsense-mediated mRNA decay. Moreover, TEC-1 significantly ameliorated the disease phenotype in an SMA murine model in vivo. Thus, TEC-1 may have promising therapeutic potential for SMA, and our study demonstrates the feasibility of RNA-targeting small-molecule drug development with an improved tolerability profile.

## Introduction

Spinal muscular atrophy (SMA) is a neurodegenerative disorder in which there is a loss of lower motor neurons (MNs) that project from the spinal cord and the brain stem. This leads to muscle atrophy and difficulties in breathing and walking, which may require tracheotomy and artificial respiration assistance.

SMA is typically inherited as an autosomal recessive trait, with most patients having loss-of-function mutations in the survival motor neuron 1 (*SMN1*) gene. The *SMN2* gene, a paralog of *SMN1*, differs from *SMN1* at only 2 base pairs in the open reading frame, but the amino acid sequences encoded by the two genes are identical. One of the base pair differences causes a translationally silent, single-nucleotide transition in *SMN2* at position 6 of exon 7 (c6t), which not only disrupts binding sites for positive splicing regulators^1^ but also creates binding sites for negative splicing regulators^2^. In addition, this change (c6t) strengthens an extended inhibitory context^3,4^. As a result, most of the mRNA transcribed from the *SMN2* gene is of the Δ7 form, which skips exon 7 via splicing. However, the full-length *SMN2* mRNA containing exon 7 (FL-*SMN2*) is also produced at a rate of 5–10% of the total transcripts. An increase in the copy number of the *SMN2* gene ameliorates the severity of SMA, and loss and compensation strongly correlate with the onset and progression of the disease^5^; hence, in recent years, *SMN2* has become an attractive target of drug development.

Nusinersen/ASO10-27/SPINRAZA, an approved antisense oligonucleotide (ASO) drug for the treatment of SMA, directly targets intronic splicing silencer N1 (ISS-N1) in intron 7 of *SMN2*^6,7^, which modulates *SMN2* splicing, and leads to an increase in SMN protein levels^8^. According to a Phase III trial (ENDEAR), nusinersen showed moderate therapeutic effects with delayed disease progression and ventilation timing^9^, but was unable to penetrate the blood–brain barrier (BBB). However, nusinersen treatment requires repeated intrathecal administration, and is associated with several side effects, including post-lumbar puncture syndrome characterized by back pain, headache, and fever, and the production of neutralizing antibodies (NAb), raising concerns associated with its clinical use^10^. Furthermore, an advanced medical procedure to treat SMA patients with scoliosis is under development, as patients with complex spinal anatomies and respiratory insufficiency are unable to receive intrathecal injections^11^. AVXS-101/ZOLGENSMA is an approved gene therapy using an adeno-associated virus serotype 9 (AAV9) to deliver a functional copy of the *SMN* gene to MNs in patients with SMA. Compared with nusinersen, clinical trials of AVXS-101 have shown that it improves survival; thus, AVXS-101 is currently regarded as the most promising drug available, provided that the first dose is administered by approximately 7 months of age^9,12^. However, there are some disadvantages of AVXS-101, such as requiring intravenous administration, and checking the presence of NAb prior to infusion^12^. In addition, ASO drugs and gene therapies are generally expensive; the costs of nusinersen for 10 years and a single treatment with AVXS-101 in the United States are approximately $4.1 and $2.1 million, respectively, which imposes an additional burden on the patients.

RG7916/risdiplam/EVRYSDI, an approved orally administered small-molecule *SMN2* splicing modulator, was developed by Roche^13,14^. Risdiplam analogs (hereafter referred to as the SMN-C series) were identified using a cell-based screening system, in which a luciferase reporter protein was expressed only when exon 7 of the *SMN2* minigene containing exons 6 to 8 was included after splicing^15^. Each of the SMN-C series included exon 7 in the *SMN2* transcript and concomitantly increased the level of FL-SMN protein in SMA patient-derived cells^15^. Optimization studies in a murine model of SMA and in non-rodent models were performed, resulting in the identification of risdiplam^16^. In the interim analysis of Phase II/III (FIREFISH), risdiplam showed equivalent activity to AVXS-101 in rescuing the motor function delays in patients with SMA type I^12,13^. However, risdiplam induced micronucleus formation in preclinical studies in vitro and in vivo, indicating that it is a potential carcinogen^16^. Aberrant splicing of forkhead box protein M1 (*FOXM1*) is thought to be responsible for this micronucleus induction^16^. In addition, RNA sequencing analyses revealed that the SMN-C series decreases the expression levels of galactosylceramidase (*GALC*)^15,17^, which metabolizes the toxic glycolipid psychosine in lysosomes^18^, and huntingtin (*HTT*)^15,17^, which is essential for pancreatic and brain function^19^. Thus, orally administered small-molecule drugs with a limited effect on splicing targets other than *SMN2* (i.e., secondary splice targets) would be the best drugs to ensure clinical tolerability.

Small-molecule compounds offer many advantages for systemic delivery, including access to the brain, heart, and skin; non-invasive (oral) administration; avoidance of NAb; and lower manufacturing costs compared to ASO and gene therapy. We identified a *SMN2* splicing small-molecule modulator, termed TEC-1 (2-(4,6-dimethylpyrazolo[1,5-*a*]pyrazin-2-yl)-6-(4-methylpiperazin-1-yl)quinazolin-4(3*H*)-one)^20^, that shows promise as a clinical candidate for the tolerable long-term treatment of SMA. This report describes the preclinical characterization and prospects of TEC-1.

## Results

### TEC-1 increases the expression level of FL-*SMN2* mRNA and decreases the expression level of Δ7 mRNA

**Figure 1:**
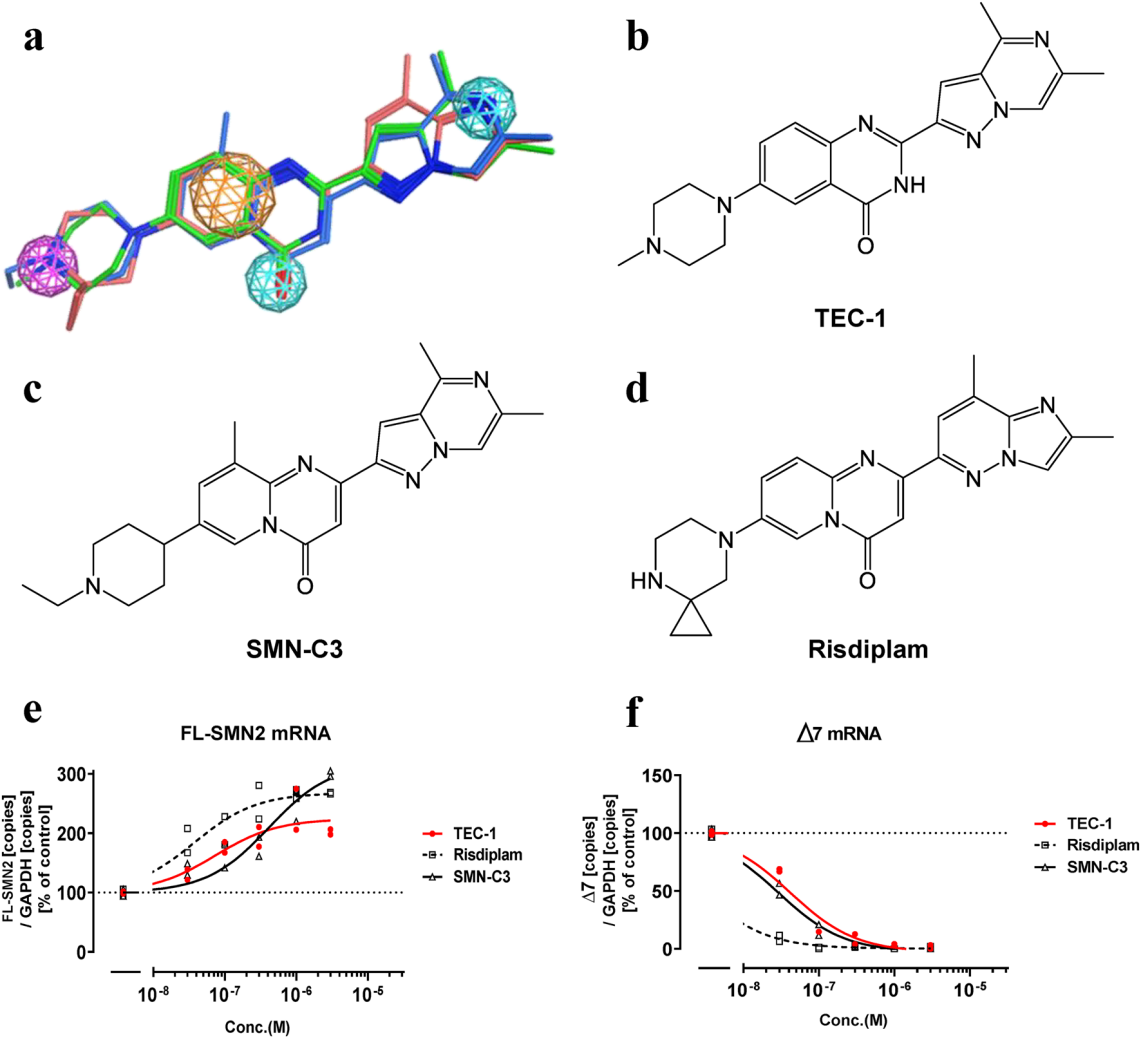
TEC-1 is structurally similar to SMN-C3 or risdiplam and modulates *SMN2* splicing. (**a**) Pharmacophore model and alignment of TEC-1 (green), risdiplam (pink), and SMN-C3 (blue) with MOE2018.01. Pharmacophore annotation: Aromatic and other pi-system rings (orange), H-bond donor heavy atom (magenta), H-bond acceptor heavy atom (pale blue). (**b**) Structure of TEC-1. (**c**) Structure of SMN-C3. (**d**) Structure of risdiplam. (**e**) qPCR analysis of FL-*SMN2* transcripts in SMA type II fibroblasts (GM03813) exposed to each compound for 24 h. The amounts of mRNA were normalized to those of *GAPDH*. (**f**) qPCR analysis of Δ7 transcripts of GM03813 cells exposed to each compound for 24 h. The amounts of mRNA were normalized to those of *GAPDH*. Data are from two biologically independent samples in e, f. a-d were derived by by Axcelead Drug Discovery Partners, Inc.

To identify splicing modulators with improved selectivity for *SMN2*, over 300 compounds were designed from our compound collection and evaluated, optimized using SMA patient-derived fibroblasts^20^. Quantitative polymerase chain reaction (qPCR) was then used to evaluate the splicing activity of these candidates against *SMN2* and secondary splice targets (e.g., *FOXM1*). Subsequently, a single oral dose was administered to FVB mice to assess bioavailability and brain penetration. As a result of these screens, we identified a compound, TEC-1, which increased the form of *SMN2* that includes exon 7 (FL-*SMN2*), and decreased the form that skips exon 7 (Δ7) in a concentration-dependent manner (Fig. 1a,b,e,f). These results led us to conclude that TEC-1 is an *SMN2* splicing modulator. The SMN-C series (SMN-C3 and risdiplam) also modulated *SMN2* splicing, with effects similar to those noted in previous reports^15,16^ (Fig. 1c–f).

### TEC-1 does not strongly affect *FOXM1* splicing

**Figure 2:**
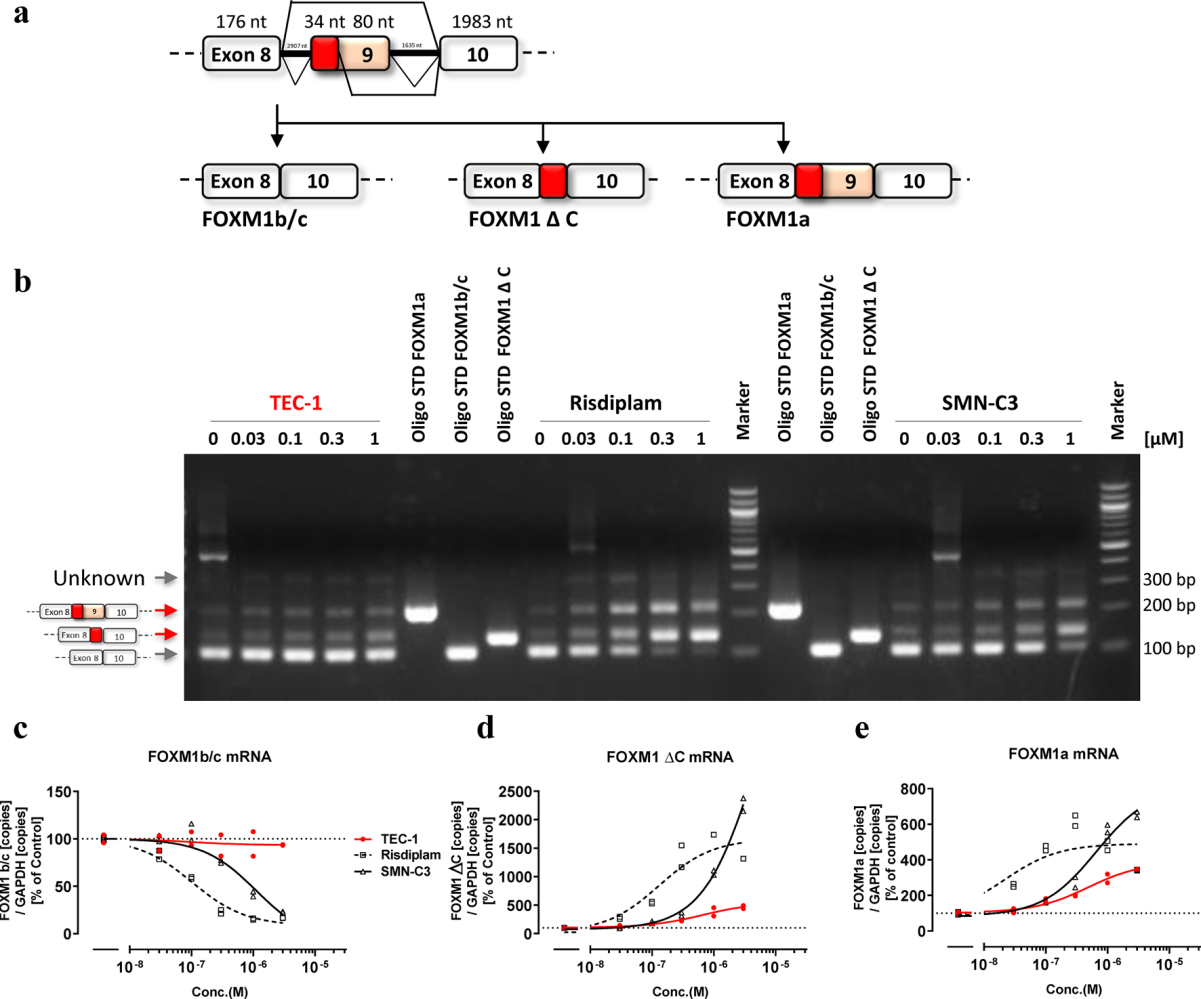
TEC-1 does not substantially affect *FOXM1* splicing. (**a**) Schematic representation of exon construction and exon inclusion of *FOXM1* mRNA splicing. (**b**) RT-PCR analysis of *FOXM1* variants of GM03813 fibroblasts exposed to each compound for 24 h. RT-PCR products of *FOXM1* variants were separated by agarose gel electrophoresis and visualized with ethidium bromide. Standard *FOXM1* fragments corresponding to three isoforms (Oligo STD) are shown. Molecular weight markers are shown on the right (bp). The full-length gel is presented in Supplementary Fig. 1e. (**c**–**e**) qPCR analysis of each *FOXM1* variant of GM03813 cells exposed to each compound for 24 h. The amounts of each variant were normalized to those of *GAPDH*. Data are from two biologically independent samples in (**c**–**e**).

FOXM1 is a protein involved in cell division, and reduced abundance of its major splicing isoform *FOXM1b/c* is observed in the G1/S phase, whereas an increase in this isoform is detected in the G2/M phase. Moreover, *FOXM1* splicing contributes to chromosome missegregation and toxicity at the level of cell cycle/division^21,22^. Previous reports revealed that risdiplam induces micronucleation in vitro and in vivo^16^. We therefore evaluated changes in *FOXM1* splicing variants that occurred under the influence of the SMN-C series by reverse transcription (RT)-PCR (Fig. 2a,b). In GM03813 fibroblasts treated with the SMN-C series, the level of the *FOXM1b/c* variant was decreased, while the levels of the *FOXM1 ΔC* and *1a* variants were increased, suggesting that the SMN-C series modulates the splicing of *FOXM1* (Fig. 2b–e). Interestingly, TEC-1 did not strongly affect the splicing of *FOXM1* (Fig. 2b–e). To estimate the selectivity of splicing between *SMN2* and *FOXM1*, we determined the EC_1.5×_ value of *SMN2*, representing the concentration at which there is a 50% increase in total FL-*SMN2* mRNA. Next, we calculated EC_50_ values of *FOXM1b/c* to determine the concentrations showing a 50% reduction in total *FOXM1b/c* mRNA levels. We then normalized EC_50_ of *FOXM1b/c* based on the EC_1.5×_ value of FL-*SMN2* (*FOXM1b*/FL-*SMN2*) and found that its value in the presence of TEC-1 was over 61 (Table 1 and Fig. 2c–e). By contrast, these values in the presence of SMN-C3 or risdiplam was 8 and 8, respectively, indicating that the SMN-C series strongly affects *FOXM1* splicing compared with TEC-1 (Table 1 and Fig. 2c–e). We next examined whether TEC-1 induces chromosomal damage using an in vitro micronucleus assay in human lymphoblasts (TK6 cells) under three treatment conditions (Table 2). In a 3-h treatment without S9 mix, followed by a 21-h recovery period, 2.3 µg/mL (5.9 µM) TEC-1 caused a significant increase in the incidence of micronucleated cells. We confirmed that TEC-1 induces a concentration-dependent increase in the number of micronucleated cells. However, the relative population doubling (RPD) value at 2.3 µg/mL (5.9 µM) was 35.0%, suggesting that this positive response was secondary to cytotoxicity. In a 3-h treatment including S9 mix, followed by a 21-h recovery period, 2.7 µg/mL (6.9 µM) TEC-1 significantly increased the incidence of micronucleated cells. The RPD value at this concentration was 58.7%. However, in this condition, a concentration-dependent increase was not observed. In a 24-h treatment without S9 mix, there was no significant increase in the number of micronucleated cells at any TEC-1 concentration compared to the negative control group. We therefore concluded that TEC-1 and its metabolites in the liver do not induce micronucleation, indicating that no chromosomal damage occurs. Conversely, risdiplam induces micronucleation in vitro and in vivo^16^. In summary, these results suggest that avoiding *FOXM1* splicing helps to prevent micronucleation.

**Table 1:** Selectivity against various splicing targets.

Comp	EC_1.5×_ (nM)	EC_50_ (nM)	EC_4.0×_ (nM)	EC_3.0×_ (nM)	EC_50_ (nM)	EC_50_ (nM)	Selectivity	Selectivity	Selectivity
	FL-SMN2	FOXM1b	FOXM1ΔC	FOXM1a	GALC	HTT	FOXM1b /FL-SMN2	GALC /FL-SMN2	HTT /FL-SMN2
TEC-1	49.1 (22.7–109.4)	> 3000.0	1311.6 (864.7–2285.8)	1096.8 (809.7–1552.2)	> 3000.0	> 3000.0	> 61	> 61	> 61
Risdiplam	15.8 (8.81–32.8)	123.5 (93.2–160.4)	38.9 (6.98–85.0)	24.2 (4.49–78.5)	N.T.	N.T.	8	N.T.	N.T.
SMN-C3	122.1 (74.5–221.7)	945.2 (620.0–1349.0)	298.5 (248.8–355.8)	280.3 (211.4–377.7)	459.4 (315.7–645.5)	547.2 (373.6–771.3)	8	4	4

**Figure 3:**
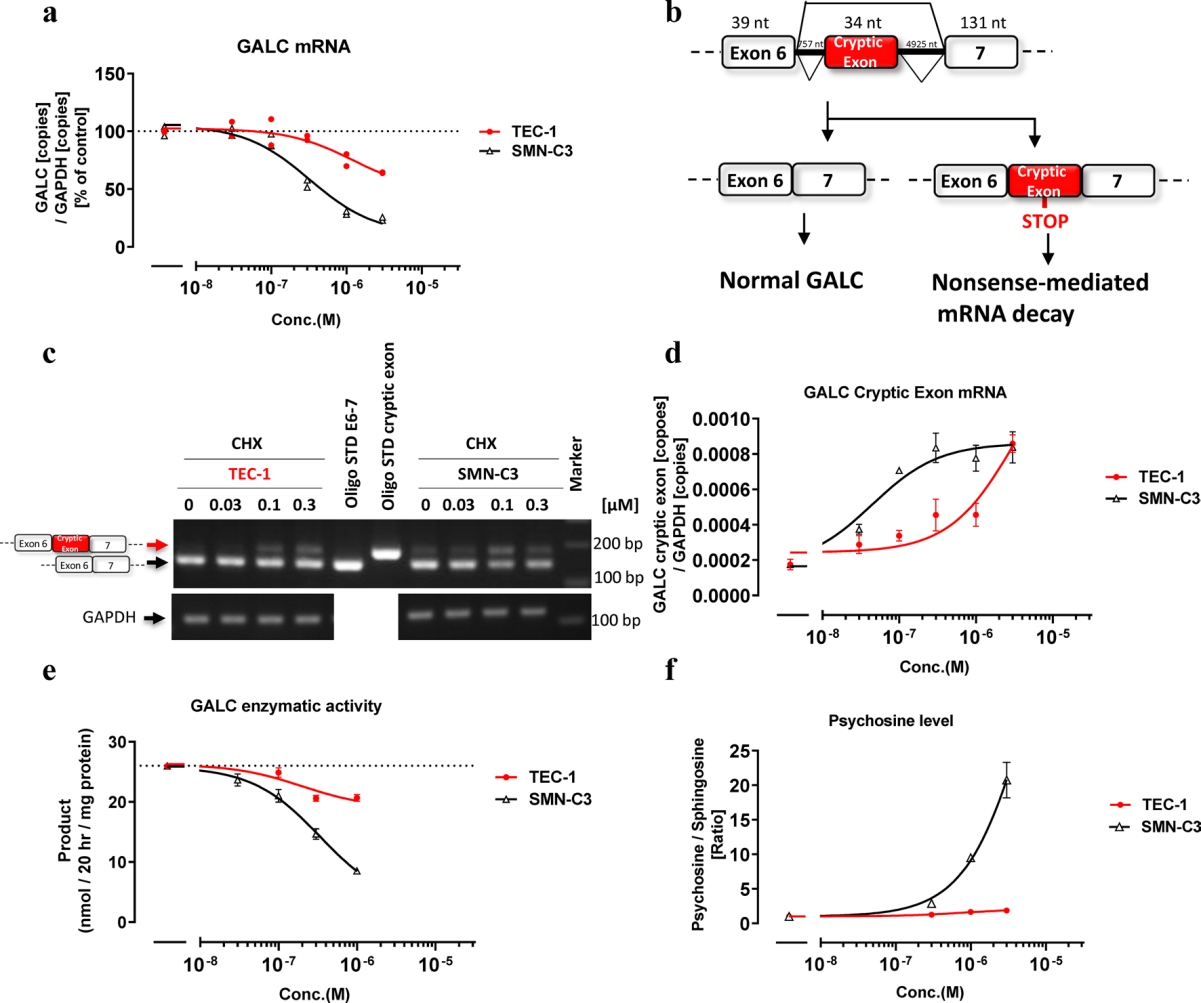
TEC-1 does not strongly impact *GALC* cryptic splicing. (**a**) qPCR analysis of *GALC* transcripts in GM03813 cells treated with each compound for 24 h. The amounts of *GALC* mRNA were normalized to those of *GAPDH*. (**b**) Schematic representation of exon construction and cryptic exon inclusion of *GALC* mRNA splicing. (**c**) RT-PCR analysis of *GALC* transcripts in GM03813 cells treated for 6 h with both compounds and CHX. RT-PCR products were separated by agarose gel electrophoresis and visualized with ethidium bromide. Standard *GALC* fragments (Oligo STD) with or without the cryptic exon are shown. Molecular weight markers are shown on the right (bp). The full-length gel is presented in Supplementary Fig. 2c. (**d**) qPCR analysis of the *GALC* cryptic exon in GM03813 cells treated for 6 h with both compounds and CHX. Aberrant *GALC* mRNA amounts with cryptic exons were quantified and normalized to those of *GAPDH*. (**e**) GALC enzymatic assay from human oligodendroglioma (Hs683) cells treated with each compound for 3 days. The amounts of standardized product (fluorogenic HMU-β-D galactopyranoside) were normalized to those of total protein. (**f**) Ratio of psychosine and sphingosine in Hs683 cells treated with each compound for 12 days. The amounts of psychosine were determined by LC/MS/MS whose signal was normalized by sphingosine signals. Data are from two biologically independent samples in (**a**). Data in (**d**), (**e**), (**f**) represent means ± standard error of the mean (SEM) of three independent assessments per concentration.

**Figure 4:**
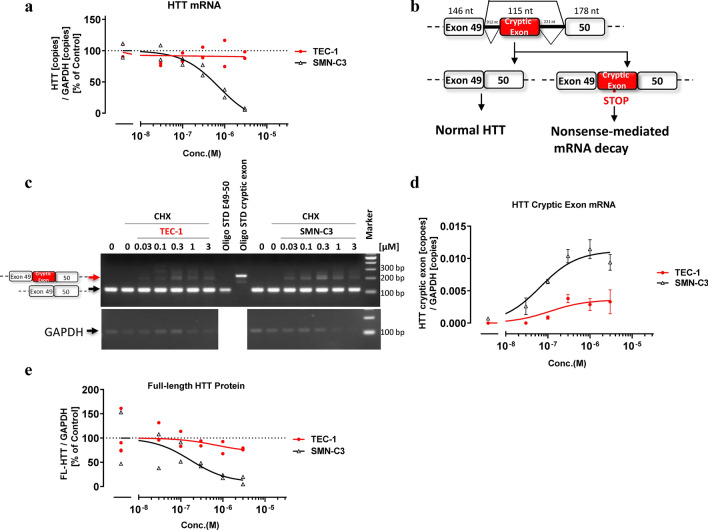
TEC-1 does not strongly affect *HTT* cryptic splicing and protein abundance unlike SMN-C3. (**a**) qPCR analysis of *HTT* transcripts in GM03813 cells treated with each compound for 24 h. The amounts of *HTT* mRNA were normalized to those of *GAPDH*. (**b**) Schematic representation of exon construction and cryptic exon inclusion or exclusion of *HTT* mRNA splicing. (**c**) RT-PCR analysis of *HTT* transcripts in GM03813 cells treated for 6 h with compounds and CHX. RT-PCR products were separated by agarose gel electrophoresis and visualized with ethidium bromide. Standard *HTT* fragments with or without the cryptic exon (Oligo STD) are shown. Molecular weight markers are shown on the right (bp). Full-length gels are presented in Supplementary Fig. 5b. (**d**) qPCR analysis of cryptic *HTT* transcripts of GM03813 cells treated for 6 h with compounds and CHX. The amounts of *HTT* with the cryptic exon were quantified and normalized to those of *GAPDH*. Values less than the quantitation limit were presumed to be 0. (**e**) Full-length (FL)-HTT protein levels of GM03813 fibroblasts treated with each compound for 3 days. Following western blot analyses, the signals of FL-HTT protein were analyzed and were normalized to those of GAPDH protein. Full-length blots are presented in Supplementary Fig. 4a. Data are from two or four biologically independent samples in (**a**), (**e**). Data in (**d**) represent means ± SEM of three independent assessments per concentration.

The EC values were calculated for a 6-point titration curve using nonlinear regression with Graphpad Prism for compounds whose curves are shown in Figs. 1, 2, 3, or 4. GM03813 fibroblasts were exposed to each compound for 24 h. The amounts of each target mRNA were normalized to those of *GAPDH*. An EC_1.5×_, _4.0×_ or _3.0×_ value shows 50%, 300%, or 200% total target mRNA increase. An EC_50_ value of the target shows 50% total target mRNA reduction. The 95% confidence intervals are listed in brackets. To estimate selectivity, each EC value was divided with EC_1.5×_ of FL-*SMN2*. N.T. indicates Not Tested.

**Table 2:** TEC-1 did not induce micronucleus formation in TK9 cells with or without a rat liver microsome fraction (S9).

Group	Test article	Concentration µg/mL	Treatment conditions	Relative population doubling (%)	Micronucleated cells			
					Well 1	Well 2	Total	(%)
1	DMSO	10 μL/mL	3 h -S9 mix	100	13	13	26	(0.65)
2	TEC-1	1.9 (4.9 µM)		68.6	13	16	29	(0.73)
3		2.1 (5.4 µM)		65.4	16	20	36	(0.9)
4		2.3 (5.9 µM)		35.0	29	27	56	(1.4) **^,##^
5	MMC	0.1 (0.3 µM)		N.D.	59	59	118	(2.95) **
6	DMSO	10 μL/mL	3 h + S9 mix	100	10	14	24	(0.6)
7	TEC-1	2.5 (6.4 µM)		76.4	18	15	33	(0.83)
8		2.7 (6.9 µM)		58.7	24	21	45	(1.13) **
9		2.9 (7.4 µM)		46.8	17	13	30	(0.75)
10	CP	6 (21 µM)		N.D.	39	46	85	(2.13) **
11	DMSO	10 μL/mL	24 h -S9 mix	100	18	12	30	(0.75)
12	TEC-1	1.1 (2.8 µM)		81.7	21	13	34	(0.85)
13		1.3 (3.3 µM)		74.4	17	12	29	(0.73)
14		1.5 (3.9 µM)		69.5	17	15	32	(0.8)
15	COL	0.01 (0.03 µM)		N.D.	45	51	96	(2.4) **

Experiments were performed under the following three treatment conditions. 1: short-term treatment for 3 h without S9 mix, followed by a 21-h culture (3 h −S9 mix, shown in groups 1–5), 2: short-term treatment for 3 h with S9 mix, followed by 21-h culture (3 h + S9 mix, shown in groups 6–10), 3: continuous treatment for 24 h without S9 mix (24 h −S9 mix, shown in group 11–15). Mitomycin (MMC) for 3 h −S9 mix, cyclophosphamide (CP) for 3 h + S9 mix, and colcemid (COL) for 24 h −S9 mix were used as positive controls. These articles have been recommended for use in the OECD test guideline (Test No. 487)^49^. DMSO was served as negative control. The numbers of micronucleated cells were counted in 4000 cells and percentages of micronucleated cells were calculated for statistical analyses. ^##^ < 0.01 as assessed by Cochran-Armitage test for dose-relationship. **p < 0.01 as assessed by Fisher’s exact test using negative control-treated cells. N.D. indicates Not Determined.

The *FOXM1* sequence in cynomolgus monkeys is nearly identical to that in humans, with only a single mismatch in the sequence between exon 9 and the donor site (Supplementary Fig. 1a, yellow highlight). Notably, *FOXM1* mis-splicing was similarly observed in cynomolgus monkey cells (NCMDF) exposed to risdiplam (Supplementary Fig. 1b–d), for which the toxicology is discussed below.

### TEC-1 only slightly modulated *GALC* splicing

SMN depletion was reported to reduce the total amount of *GALC* mRNA in mouse MNs^23^. The SMN-C series, which increases the amount of SMN protein, unexpectedly decreased the total amount of *GALC* mRNA in RNA sequencing studies of SMA patient-derived fibroblasts^15,17^. We speculated that this reduced *GALC* expression is caused by the effects of the SMN-C series on secondary splice targets, rather than by an effect mediated by the SMN protein itself. To test this possibility, we evaluated the ability of SMN-C3 or TEC-1 to change the expression of *GALC* mRNA. SMN-C3 strongly reduced the *GALC* mRNA level compared to TEC-1 (Fig. 3a). The EC_50_ values of *GALC*, which reflects a 50% reduction in total *GALC* mRNA, normalized to the EC_1.5×_value of FL-*SMN2* (*GALC*/FL-*SMN2*) was over 61 for TEC-1, whereas that of SMN-C3 was 4, suggesting that TEC-1 is a more selective *SMN2* splicing modulator compared with the SMN-C series (Table 1, Fig. 3a). To investigate the mechanism of action for the reduction in *GALC* mRNA expression induced by the SMN-C series, we assessed whether the decrease in *GALC* mRNA by SMN-C3 is affected by cycloheximide (CHX), which suppresses nonsense-mediated mRNA decay (NMD). The *GALC* mRNA reduction was rescued by CHX exposure (Supplementary Fig. 2a). Direct sequencing of RT-PCR products revealed that a 34 nucleotide (nt) sequence derived from intron 6 was inserted between exons 6 and 7 (Supplementary Fig. 2b cryptic exon start, Fig. 3b). Thus, we confirmed a previously unreported 34 nt cryptic exon by RT-PCR and qPCR, the inclusion of which was more significantly induced by SMN-C3 than by TEC-1 (Fig. 3c,d). This cryptic exon inclusion in *GALC* causes NMD since an in-frame TAG codon (premature termination codon) exists 12 nt from the 5ʹ-terminus of the cryptic exon (22 nt from the 3ʹ end of the cryptic exon) (Supplementary Fig. 3a, highlighted green). Thus, we conclude that the SMN-C series promotes inclusion of this abnormal 34 nt cryptic exon, which contributes to the reduction in total *GALC* mRNA expression.

Notably, reductions in *GALC* by the SMN-C series were not observed in cells derived from dogs and rats, whose genomes lack the long intronic region that includes the 34 nt sequence (Supplementary Fig. 3a, d, e). However, the reduction in *GALC* caused by the SMN-C series in cynomolgus monkey cells was stronger than that in green monkey cells (Supplementary Fig. 3a–c). This difference is likely related to the number of mismatched sequences near the cryptic exon and donor site (Supplementary Fig. 3a–c). Furthermore, the sequences of the human *GALC* cryptic exon and human *FOXM1* exon 9/A2 are quite similar (only 3 mismatches in the included exon and donor site, Supplementary Fig. 11, highlighted yellow). These results suggest that sequence similarity contributes to the inclusion of the *GALC* cryptic exon by the SMN-C series.

*GALC* encodes a lysosomal enzyme that degrades psychosine, a highly toxic glycolipid^18^. Mutations in *GALC* underlie Krabbe disease, an autosomal recessive disorder in which psychosine accumulates in the brain. To test whether reduced *GALC* mRNA impacts GALC enzymatic function, the activity of GALC protein was measured. Expression of UDP glycosyltransferase 8 (UGT8), a key enzyme for the production of psychosine, is low in the skin^24^. Since UGT8 expression is high in oligodendrocytes^25^, its glioma cell line Hs683 was utilized for this assay. SMN-C3 strongly decreased the activity of GALC compared to TEC-1, similar to its effects on *GALC* mRNA expression (Fig. 3a,e). TEC-1, even at a concentration of 3000 nM, induced a slight decrease in GALC enzymatic activity, and caused only a 1.9-fold increase in psychosine compared to the control (Fig. 3e,f). Notably, a 1.9-fold increase in psychosine is tolerable, as heterozygous carriers of Krabbe disease have an approximately 1.8-fold increase in plasma psychosine compared with unaffected controls^18^. By contrast, 3000 nM SMN-C3 triggered an approximately 22-fold increase in psychosine (Fig. 3f), which is greater than the approximately 15-fold increase in plasma psychosine reported in early-infantile-onset Krabbe disease^18^.

### TEC-1 does not induce abnormal *HTT* splicing

Knockdown of the *HTT* gene in young mice was reported to cause acute pancreatitis^19^. Previous RNA sequencing studies have reported that the SMN-C series reduces total *HTT* mRNA^15,17^, which we confirmed in the present study using qPCR (Fig. 4a). However, SMN depletion does not alter the level of *HTT* mRNA in mouse MNs^23^, suggesting that *HTT* reduction by the SMN-C series is caused by secondary splice target effects. To elucidate the mechanism by which *HTT* is reduced, RT-PCR products from cells cultured with both risdiplam and CHX were sequenced. In the presence of risdiplam with CHX, the *HTT* mRNA included a previously unreported 115 nt derived from part of intron 49, just after the end of exon 49 (Fig. 4b, Supplementary Fig. 4b), which we also confirmed by RT-PCR and qPCR (Fig. 4c,d). As in the case with the *GALC* splicing isoforms, an in-frame TAG codon (premature termination codon) was present 58 nt upstream from the 3ʹ end of the cryptic exon (57 nt from the 5ʹ end) (Supplementary Fig. 5a, highlighted green). These results suggest that the *HTT* mRNA with the cryptic exon was also degraded via NMD. Importantly, TEC-1 did not substantially impact the inclusion of the *HTT* cryptic exon or reduction of *HTT* at the mRNA and full-length protein levels (Fig. 4a,d,e).

### TEC-1 modulates *SMN2* splicing and shows disease-modifying effects in induced pluripotent stem cell (iPSC)-derived MNs of an SMA patient

**Figure 5:**
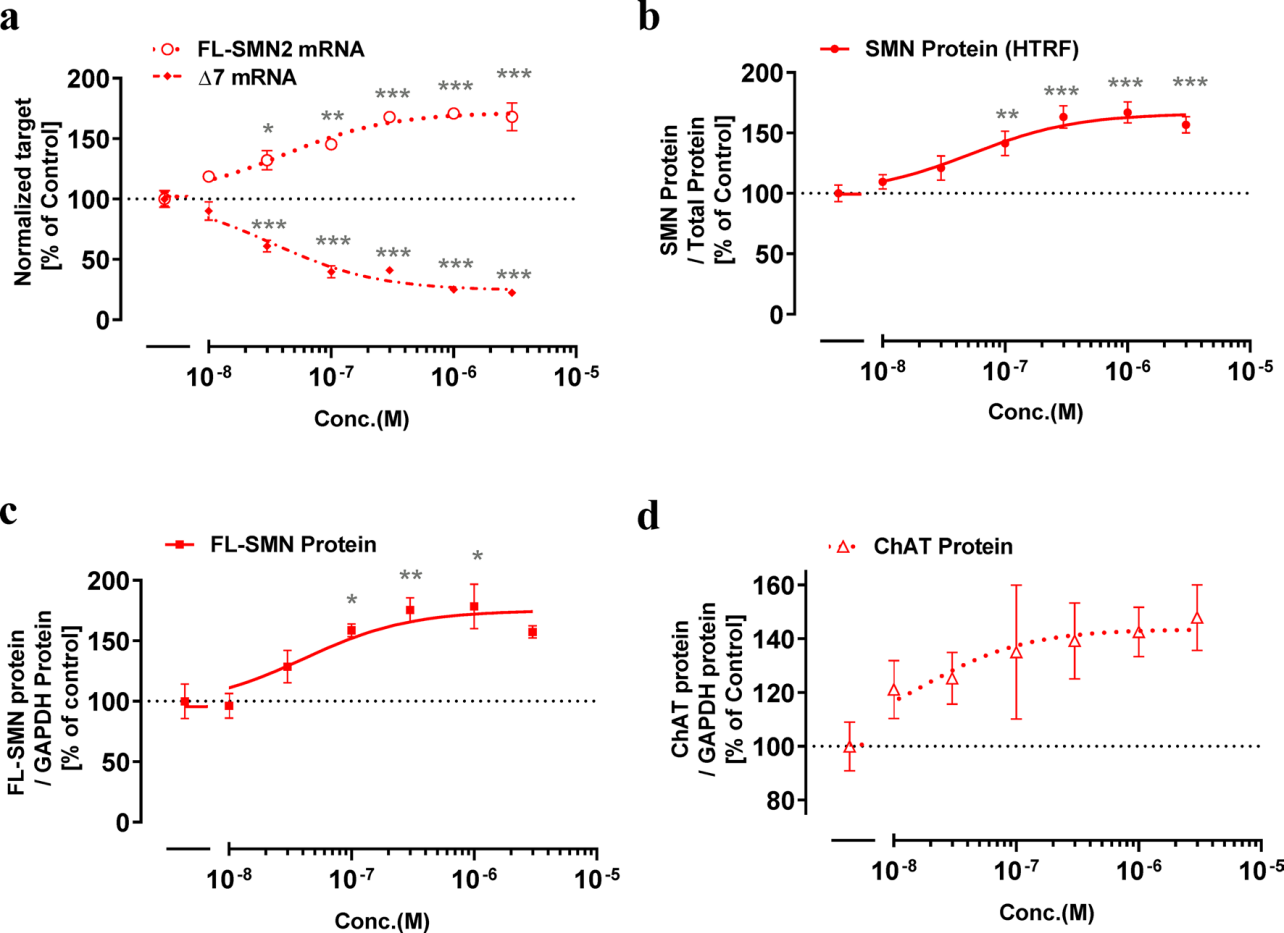
TEC-1 displays disease-modifying effects in motor neurons derived from SMA patient iPSCs (GM24468). (**a**) qPCR analysis of the *SMN* transcripts of motor neurons treated with TEC-1 for 24 h. The amounts of mRNA, including (FL-*SMN2*), and excluding (Δ7) exon 7, were normalized to those of *GAPDH*. (**b**) SMN protein levels in motor neurons treated with TEC-1 for 3 days. SMN protein in cell lysates was quantified with homogeneous time resolved fluorescence (HTRF) and normalized to total protein. (**c**) The level of FL-SMN protein. The SMN density in western blots was normalized to GAPDH density. Full-length blots are presented in Supplementary Fig. 10. (**e**) The levels of ChAT protein. The ChAT density in western blots was normalized to GAPDH density. Full-length blots are presented in Supplementary Fig. 10. Data in (**a**–**d**) represent means ± SEM of three independent assessments per concentration. *p < 0.05, **p < 0.01, ***p < 0.001 as assessed by one-way ANOVA followed by Dunnett’s test using DMSO-treated cells as a control (100%).

To test whether TEC-1 impacts *SMN2* splicing in MNs, we cultured MNs differentiated from iPSCs derived from a patient with SMA type II^26^. TEC-1 showed clear ability to modulate *SMN2* splicing at a concentration of 30 nM, and increased SMA protein at a concentration of 100 nM (Fig. 5a–c). Consistent with previous results, we found that SMN-C3 modulates *SMN2* splicing in our culture conditions^15^, with similar effects to those of TEC-1 (Supplementary Fig. 6). TEC-1 has also been shown to increase the levels of choline acetyltransferase (ChAT), which are decreased in MNs from patients with SMA compared to those of healthy subjects^27^ (Fig. 5d). These results suggest that TEC-1 can modulate *SMN2* splicing in MNs, and is therefore a potential therapeutic drug.

To ensure its therapeutic benefit to SMA patients in combination with nusinersen, it is important to know whether TEC-1 inhibits the activity of nusinersen. Low concentrations of TEC-1 did not inhibit the effects of nusinersen in SMA patient-derived fibroblasts, whereas at 1 and 3 µM, it significantly enhanced the effects of nusinersen (Supplementary Fig. 7).

### TEC-1 ameliorates the disease phenotype in a murine model of SMA

**Figure 6:**
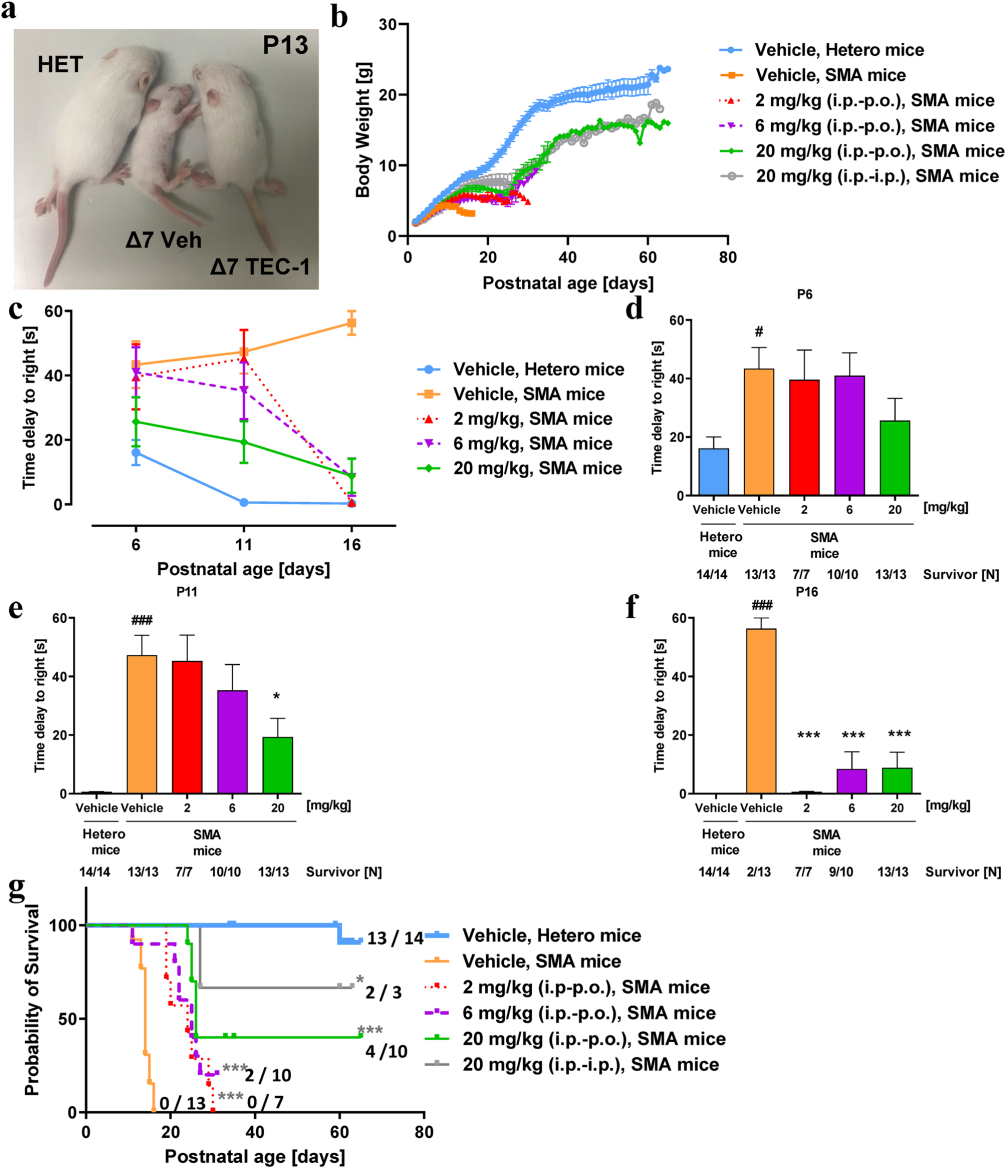
TEC-1 rescues the phenotype in a murine model of SMA. (**a**) Appearance of a vehicle-treated SMA mouse model, a 20 mg/kg TEC-1-treated SMA mouse model, and a vehicle-treated heterozygous mouse (HET) at P13. The murine model of SMA was treated from P2 to P23 once daily with vehicle or TEC-1 by intraperitoneal administration (i.p.) at 2, 6, or 20 mg/kg, and thereafter once daily by oral administration (p.o.) or i.p. with 2, 6, or 20 mg/kg. (**b**) Body weight from P2 through P65. (**c**–**f**) Righting reflex of SMA mice. The mean time for mice to right themselves after being put onto their backs, assessed at P6, P11, and P16, total duration. ^#^*p* < 0.05, ^##^*p* < 0.01 and ^###^*p* < 0.001 (vs. HET/Hetero, vehicle) as assessed by Student’s t-test (unpaired, two-tailed). *p < 0.05, **p < 0.01 and ***p < 0.001 as assessed by one-way ANOVA followed by Dunnett’s test using vehicle-treated SMA mice as a control. Individuals who died had a value of 60 s (maximum). The number of mice (N) is shown in the graph. (**g**) Kaplan–Meier survival curves from birth to P65. Numbers at the right indicate survivors at P65 mice per group. The log-rank (Mantel-Cox) test and Bonferroni test were used for comparisons. *p < 0.05, **p < 0.01, ***p < 0.001 (vs. vehicle [KO]). The survivor or total input number of mice is graphed.

To examine whether TEC-1 alleviates the SMA phenotype, we evaluated the effects of TEC-1 on a murine model of SMA (SMNΔ7 mice), which show a severe disease phenotype^28^. First, we confirmed that orally administered TEC-1 could penetrate the BBB in adult wild-type FVB mice (Supplementary Fig. 8 and Supplementary Table 4). Next, since oral administration to neonatal SMNΔ7 mice increases the rate of unexpected mortality^29^, we intraperitoneally administered TEC-1 before weaning (P2 to P23). Intraperitoneally administered TEC-1 was absorbed, excreted, and crossed the BBB in juvenile wild-type FVB mice (Supplementary Fig. 9 and Supplementary Table 5). SMNΔ7 mice were then treated with 2, 6, and 20 mg/kg TEC-1 once a day by intraperitoneal injection from P2 to P23. After P23, the route of administration was changed to oral administration, since oral drug development is the ultimate objective. SMNΔ7 mice treated with TEC-1 showed an increased body mass compared to that of vehicle-treated SMNΔ7 mice (Fig. 6a,b). To examine the effect of TEC-1 on motor function in SMNΔ7 mice, a righting reflex test was performed at P6, P11, and P16. Latency to righting in vehicle-treated SMNΔ7 mice was prolonged compared to that of control heterozygous mice. However, SMNΔ7 mice treated with 2 mg/kg TEC-1 showed a significantly shortened latency time compared to that of vehicle-treated mice at P16 (Fig. 6c–f). Importantly, TEC-1 enhanced the survival of SMNΔ7 mice compared with that of vehicle-treated SMNΔ7 mice (Fig. 6g). Taken together, TEC-1 has potential to ameliorate SMA phenotypes, improving both survival and motor function.

## Discussion

TEC-1 was identified and optimized using a cell-based screening system with fibroblasts from a patient with SMA, in contrast to the previous use of a cell-based assay using an *SMN2* minigene reporter, which led to the identification of the SMN-C series^15^ and the NVS-SM series^30^. To improve the clinical tolerability, we simultaneously evaluated the splicing of *SMN2* and secondary splice targets (e.g., *FOXM1*) in SMA patient-derived fibroblasts, which maintain the intrinsic structure of cellular mRNAs. Our approach confirmed that TEC-1 has improved selectivity toward *SMN2* splicing over three representative secondary splice targets. The characterization of the molecular target or binding site of TEC-1 is beyond the scope of this study. Previous reports revealed that the SMN-C series and NVS-SM series directly interact with the major groove of the RNA duplex generated by the 5ʹ splicing site of exon 7 and U1 snRNA^17^. Furthermore, the SMN-C series also binds to purine-rich regions within exon 7, and this interaction is proposed to be affected by several RNA-binding protein factors^17,31,32^. Thus, multiple interactions of RNA structure and compounds contribute to selective *SMN2* splicing by the SMN-C series. Further research is required to reveal that TEC-1, which has a similar pharmacophore to the SMN-C series (Fig. 1), interacts in the same manner and strongly and/or selectively binds to *SMN2* over the three secondary splice targets compared with the SMN-C series. Secondary splice target effects may lead to a high risk of toxicity in vivo. Indeed, in a 39-week toxicological study of cynomolgus monkeys treated with risdiplam, pathological intestinal and pancreatic changes, and irreversible retinal degeneration were observed^16^. Ratni et al.^16^ hypothesized that *FOXM1* may be the most important target contributing to these toxicities, although the involvement of other secondary splice targets such as *STRN3*, *APLP2*, and *MADD* remains unclear. Our in vitro analysis of cynomolgus monkey cells also suggested that aberrant splicing of *FOXM1* contributes to the toxicity in risdiplam-treated cynomolgus monkeys (Supplementary Fig. 1). To further characterize the selectivity of splicing by TEC-1, in future studies, we plan to investigate not only these secondary splice targets (*STRN3*, *APLP2*, and *MADD*) but also other unexpected targets using RNA sequencing and qPCR. Roche/PTC, when developing risdiplam, had set an exposure cap in a Phase I clinical trial with its concentration showing an area under the curve (AUC)_0–24h_ value of 1500 ng·h/mL in plasma (18 mg/individual)^14^. Risdiplam administered under this exposure cap showed a comparable clinical effect to AVXS-101 in motor function assessments. Only about half of the patients achieved a clinical readout, which was the ability to sit unsupported for several seconds (5 or 30 s) during interim analysis^12,13^. Therefore, there is still room to improve motor function delays. Based on these reports and our results, we believe that risdiplam was being tested at lower doses in clinical trials to avoid toxic effects such as *FOXM1* or *GALC* splicing and micronucleus induction^16^. Risdiplam aberrantly included *FOXM1* exon 9, whose nucleotide sequence is similar to an unreported *GALC* cryptic exon (Supplementary Fig. 11, highlighted yellow). We hypothesize that the secondary splice target effects of the SMN-C series stem from these conserved nucleotide sequences of RNA. By contrast, TEC-1 showed relatively higher selectivity against *FOXM1*, *GALC,* or *HTT*. Our screening strategy with cells which maintain the intrinsic structure of cellular mRNAs further proved the validity of excluding toxic cryptic exons in specific secondary splice targets, enabling the identification of small-molecule *SMN2* splicing modulators with a more tolerable clinical profile.

The biological activity of 106 representative proteins that are drug discovery targets was investigated with a selectivity-panel from Eurofins Inc. TEC-1 weakly affected the biological activity of only two targets, human acetylcholinesterase and rat N type calcium channel, with IC_50_ values of 1.19 μM and 8.80 μM, respectively. TEC-1 is therefore an effective and safe drug since the EC_1.5×_ value of FL-*SMN2* (49.1 nM) is markedly lower than the two aforementioned IC_50_ values. Furthermore, TEC-1 showed good profiles with general in vitro toxicity and adsorption, distribution, metabolism, and excretion (ADME) tests (items: solubility, PAMPA/membrane permeability; MDR1/BBB penetration, stability; CYP inhibition, CYP induction; PXR, hERG, cytotoxicity/liver toxicity, umu/mutagenicity, Ames/mutagenicity, in vitro micronucleus test/tumorigenesis). It should be noted that TEC-1 did not induce micronucleus formation in human cells in vitro, in contrast to risdiplam. Collectively, our findings demonstrate that TEC-1 is a key member of a class of compounds with a low risk of acute and chronic side effects for SMA treatment.

Cardiac abnormalities^33,34^, pancreatic defects^35,36^, and liver deficits^37,38^ have been reported in patients with SMA and in the murine model of SMA. Small-molecule compounds delivered systemically have the potential to increase the expression of SMN proteins, which are expressed ubiquitously in humans. Pinna and tail necrosis have been reported in the SMA murine model treated with SMN gene therapy^39^ or low doses of nusinersen^40^, which may result from insufficient systemic delivery or the impact of large-molecule drugs. Long-term clinical observations of these therapeutics are therefore recommended^41,42^. The small-molecule compound TEC-1 is expected to be a more promising drug for the long-term treatment of patients with SMA compared with the two approved large-molecule drugs whose systemic delivery is limited. From this single pharmacokinetics studies in juvenile (intraperitoneal) and adult (oral) FVB mice, the Kp values, which indicate the ratio of the compound in the brain and plasma, were greater than 1 (Supplementary Figs. 8 and 9). Thus, TEC-1 was efficiently distributed to the brain in both juveniles and adults, suggesting a favorable systemic delivery that includes the brain. Although the drug-metabolizing enzyme of TEC-1 has not yet been identified, TEC-1 was excreted from the plasma and brain in a time-dependent manner in juvenile mice. This indicates that TEC-1 can be developed for use in juvenile patients whose compositions of drug-metabolizing enzymes are different from those of adults. All SMA model mice administered 20 mg/kg TEC-1 intraperitoneally survived until weaning. TEC-1 was absorbed from the peripheral circulation, crossed the BBB, and completely rescued SMA phenotypes in vivo until weaning age. Interestingly, 66% (2 of 3 mice) of the SMA mice administered TEC-1 intraperitoneally survived, whereas 40% (4 of 10 mice) of SMA mice receiving oral administration survived, suggesting that bioavailability of the drug administered intraperitoneally is more favorable than when administered orally after weaning. To improve oral bioavailability, new formulation technologies^43^ will be applied to TEC-1 for the development of SMA therapeutics. At this time, the TEC-1 dosage is higher than that of risdiplam to extend the lifespan in the SMA murine model^16^. Finally, we will examine whether formulated TEC-1 increases the oral bioavailability, and enhances the exposure cap and therapeutic effects compared with risdiplam for SMA in preclinical and clinical studies.

In conclusion, these findings indicate that TEC-1 has selectivity toward *SMN2* splicing over three secondary splice targets, suggesting that TEC-1 is a disease-modifying drug with a potentially higher therapeutic window compared to the SMN-C series, including risdiplam. Furthermore, TEC-1 did not inhibit the action of nusinersen in a cell culture system, supporting the possibility that TEC-1 could be utilized concomitantly with this existing SMA drug. Identification of TEC-1 contributes not only to the development of a promising SMA therapeutic but also to the feasibility of RNA-targeting small-molecule drug discovery that ensures clinical tolerability.

## Materials and methods

### *SMN2* splicing modulators

RBS-001/TEC-1 (2-(4,6-dimethylpyrazolo[1,5-*a*]pyrazin-2-yl)-6-(4-methylpiperazin-1-yl)quinazolin-4(3*H*)-one) is a novel, orally available *SMN2* splicing modifier that favors the production of full length *SMN2* mRNA with high selectivity^20^. Risdiplam and the analogue, SMN-C3, were synthesized in-house according to the literature^15,16^. An antisense oligonucleotide (ASO) with the same sequence and modification as nusinersen was obtained from Gene Design Inc. (Japan).

### Oligonucleotides for qRCR, RT-PCR

Oligo DNA standards, primers and probes were synthesized by Integrated DNA Technologies Inc. (USA). Their sequences are also available in Supplementary Tables 1, 2 and 3. The primer set and probe of human GAPDH, ready-made 20 × gene expression PCR assay [Cat.No.4326317E, Hs99999905_m1], was purchased from Thermo Fisher Scientific (USA). Sequences of oligo DNA standards are shown in Supplemental Table 3.

### Cell culture

Unless described otherwise, all reagents were purchased from Thermo Fisher Scientific. Both primary fibroblast cells (GM03813), collected from a SMA type II patient and induced pluripotent stem cells (iPSCs) (GM24468) derived from GM03813, were obtained from Coriell Cell Repositories (USA). GM03813 cells was cultured in Dulbecco’s Modified Eagle Medium (DMEM), high glucose containing 4.5 g/L glucose, 4 mM L-glutamine and 10% (by volume) fetal bovine serum (FBS). GM24468 cells was cultured in Essential 8 medium containing 3 µM Y-27632. GM24468 were dissociated with 0.5 × TrypLE Select solution. Hs683, human oligodendroglioma cell line, was purchased from the American Type Culture Collection (USA) and maintained in DMEM, high glucose with 10% FBS. NRK-49F, rat kidney cell line, was cultured in DMEM (Wako, Japan) with 4.5 g/L glucose, 1 × GlutaMax, 1 × Non-Essential Amino Acids and 5% FBS. Madin-Darby Canine Kidney (MDCK) and Normal African Green Monkey Kidney Fibroblast Cells (CV-1) were maintained in Eagle’s Minimum Essential Medium (Wako) with 1 g/L glucose, 1 × GlutaMax and 10% FBS. Normal Cynomolgus Monkey Dermal Fibroblast (NCMDF) was cultured in DMEM (Wako) with 4.5 g/L glucose, 1 × GlutaMax and 20% FBS. NRK-49F, MDCK, CV-1 and NCMDF cells were obtained from the Japanese Collection of Research Bioresources (Japan). Human lymphoblastoid-derived TK6 cells were purchased from American Type Culture Collection (USA) and cultured in the culture medium consisted of RPMI1640 supplemented with 10% heat-inactivated horse serum, 2 mmol/L sodium pyruvate, 100 unit/mL penicillin and 100 µg/mL streptomycin. All above cells were kept in a 37 °C incubator supplied with 5% CO_2_.

### Differentiation of iPSCs to motor neuron

GM24468 cells were differentiated to motor neurons (MNs) via a motor neuron progenitor (MNP) as previously reported^26^. Briefly described, GM24468 were treated with a chemically defined neural medium, including DMEM/F12, Neurobasal medium at 1:1, 0.5% N2, 0.1% B27, 0.1 mM ascorbic acid (Sigma Aldrich, USA), 1 × GlutaMax, and 1 × penicillin/streptomycin. 3 µM CHIR99021 (Wako), 2 µM DMH1 (Sigma Aldrich) and 2 µM SB431542 (Wako) were added in the medium. The culture medium was changed every other day. GM24468 maintained under this condition for 6 days were induced into neuroepithelial progenitors (NEPs). NEPs were then dissociated and split with dispase (Stemcell technologies) and neural medium containing 0.1 µM RA (Sigma Aldrich), 0.5 µM Purmorphamine (Pur) (Merck, USA), 1 µM CHIR99021, 2 µM DMH1 and 2 µM SB431542. The medium was changed every other day. NEPs maintained under this condition for 6 days differentiated into OLIG2^+^ MNPs. The OLIG2^+^ MNPs were expanded with the same medium containing 1 µM CHIR99021, 2 µM DMH1, 2 µM SB431542, 0.1 µM RA, and 0.5 µM Pur, and split once a week. To induce MNs differentiation, OLIG2^+^ MNPs cultured in suspension in the above neural medium with 0.5 µM RA and 0.1 µM Pur. The medium was changed every other day. OLIG2^+^ MNPs under this condition for 6 days differentiated into MNX1^+^ MNs. The MNX1^+^ MNs were then dissociated into single cells and plated on Matrigel-coated plates. The MNX1^+^ MNs were cultured with 0.5 µM RA, 0.1 µM Pur and 0.1 µM Compound E (Calbiochem, USA) for 10 days to mature into CHAT^+^ MNs.

### Transfection of ASO into cells

Transient transfection of GM03813 fibroblasts with nusinersen (final concentration 100 nM) was performed using Lipofectamine 2000. Cells were seeded 6 h prior to transfection at a density of 5 × 10^3^ cells/well in 96-well plate. Transfection was performed according to manufacturer’s instructions. Cells were co-incubated either 0.1% DMSO or with TEC-1 at the concentration of 0.03, 0.1, 0.3, 1, and 3 µM for 24 h.

### qPCR

GM03813 fibroblasts, GM24468-derived MNs, NRK-49F, MDCK, CV-1, or NCMDF cells were plated at a density of 0.5 or 1.5 × 10^4^, 4 × 10^4^, 5 × 10^3^, 5 × 10^3^, 8 × 10^3^, or 6 × 10^3^ cells/well on 96-well plates, respectively. After approximately 6 h, the medium was added with the same volume of culture medium containing each compound at the concentration of 0.06, 0.2, 0.6, 0.2, and 6 µM. The final concentration of DMSO was adjusted to 0.1% in culture condition.

All cells were treated with above compounds for 24 h and washed once with Dulbecco’s Phosphate-Buffered Saline without Ca and Mg [D-PBS (–)] (Wako or Nakarai Tesque). In case of using GM03813 or GM24468-derived MNs, cell lysis and reverse transcription were carried out using SuperPrep Cell Lysis & RT Kit for qPCR in accordance with manufacturer’s recommendations (Toyobo, Japan). qPCR was performed using THUNDERBIRD Probe qPCR Mix (Toyobo). The final concentrations of primers and probes were adjusted to 0.4 µM and 0.15 µM, respectively (Supplementary Table 1). Cycle conditions were as follows; 95 °C for 1 min; followed by 40 cycles of denaturation at 95 °C for 15 s; annealing and elongation at 60 °C for 1 min in the ViiA7 RT-PCR system (Thermo Fisher Scientific). The copy numbers of each target were normalized to those of *GAPDH*.

In the instances of using NRK-49F, MDCK, CV-1 or NCMDF, cell lysis and reverse transcription were carried out using Custom Cells to CT Lysis Components (Ambion) and Cells-to-CT RT Components (Ambion), each in accordance with manufacturer’s recommendations. qPCR was conducted using TaqMan Fast Advanced Master Mix. The final concentrations of primers and probes were adjusted to 0.4 µM and 0.15 µM, respectively. Cycle conditions were as follows; 50 °C for 2 min and 95 °C for 2 s; followed by 40 cycles of denaturation at 95 °C for 1 s; annealing and elongation at 60 °C for 20 s in the ABI7900HT RT-PCR system (Applied Biosystems, USA). The relative quantification in gene expression was determined using the 2−ΔΔCt method.

### Analysis of full length GALC with or without CHX

In order to elucidate the NMD of *GALC* transcripts, Hs683 cells were seeded at 5 × 10^3^ cells/well in 96-well plates. After approximately 6 h, cells were co-incubated with final concentration of 200 µg/ml cycloheximide (CHX) and each compound (1 µM TEC-1 or 1 µM SMN-C3, 0.1% DMSO) for 6 h.

Total RNA was extracted with RNeasy Mini kit and RNase-Free DNase Set (Qiagen, USA) from compound-treated cells, followed by cDNA synthesis using High capacity cDNA revere transcription kit (Applied Biosystems) according to their protocols. PCR was done with PrimeStar GXL DNA polymerase (Takara). Briefly, cDNA of *GALC* coding full length sequence was amplified by PCR using the primer set, 5ʹ-GAGTCATGTGACCCACACAATG-3ʹ and 5ʹ-GAATGTTAGGGAACACACCAGGTA-3ʹ. Cycle conditions for *GALC* transcripts were used as follows; 98 °C for 1 min followed by 35 cycles of denaturation at 98 °C for 10 s; annealing at 65 °C for 10 s; elongation at 72 °C for 4 min, and a final incubation at 72 °C for 5 min. The products were analyzed with agarose gel electrophoresis.

### RT-PCR of cryptic exons of GALC, HTT and FOXM1

In order to elucidate splicing pattern of cryptic *GALC* and *HTT* transcripts, GM03813 cells were seeded at 5 or 15 × 10^3^ cells/well in 96-well plates and incubated with TEC-1 or SMN-C3 (0.03, 0.1, 0.3, 0.1, 1 or 3 µM) for 6 h under final concentration of 200 µg/ml CHX. For *FOXM1*, GM03813 fibroblasts were plated at 1.5 × 10^4^ cells/well in 96-well plate and incubated with TEC-1, SMN-C3 or risdiplam (0.03, 0.1, 0.3 and 1 µM) for 24 h.

cDNA synthesis was carried out using SuperPrep Cell Lysis & RT Kit for qPCR (Toyobo, Japan). PCR amplification was carried out using Takara Ex Taq Hot Start (Takara). The final concentrations of primers was adjusted to 1 µM (Supplementary Table 2). Cycle condition was as follows; 98 °C for 1 min followed by 45 cycles of denaturation at 98 °C for 10 s; annealing at 65 °C for 10 s; elongation at 72 °C for 1 min. PCR products were separated with electrophoresis on a 3% agarose gel and visualized after ethidium bromide staining under UV light.

### Analysis of cryptic GALC and HTT transcripts

Hs683 cells which were co-treated with 1 µM SMN-C3 and 200 µg/ml CHX for 6 h were used for analysis of *GALC* transcript, while Hs683 cells which co-treated with 0.3 µM risdiplam and 200 µg/ml CHX for 6 h were used for the analysis of *HTT*.

Total RNA was extracted with RNeasy Mini kit and RNase-Free DNase Set from compound-treated cells, followed by cDNA synthesis using High capacity cDNA revere transcription kit according to their protocols. PCR was done with PrimeStar GXL DNA polymerase. Briefly, cDNA containing from exon 5 to exon 7 of *GALC* coding sequence was amplified by PCR using the primer set, 5ʹ-GAGTCATGTGACCCACACAATG-3ʹ and 5ʹ-GAATGTTAGGGAACACACCAGGTA-3ʹ. Cycle conditions for *GALC* transcripts were used as follows; 98 °C for 1 min followed by 35 cycles of denaturation at 98 °C for 10 s; annealing at 65 °C for 10 s; elongation at 72 °C for 4 min, and a final incubation at 72 °C for 5 min. The products were analyzed with 1% agarose gel electrophoresis. The second PCR was done using the first PCR products as templates with the same conditions but for only 25 cycles. One of the amplified products, derived from the cells treated with the compound, was sequenced directly using a primer, 5ʹ-CACAATGGCTGAGTGGCTACTC-3ʹ and Big Dye terminator v3.1 cycle sequencing kit. For analysis of the *HTT* transcripts, cDNA sequence from exon 42 to exon 55 was amplified by PCR using the primer set, 5ʹ-GAGGATTCTGACTTGGCAGCCA-3ʹ and 5ʹ-CACAGGCACAGTCATTGCACTGA-3ʹ with next condition; 98 °C for 1 min followed by 35 cycles of denaturation at 98 °C for 10 s; annealing at 65 °C for 10 s; elongation at 72 °C for 2 min, with a final incubation at 72 °C for 4 min. The second PCR was performed using the first PCR product as a template under the same conditions but only for 20 cycles. The amplified product was sequenced directly using a primer, 5ʹ-ATGAATGCCTTCATGATGAACTCG-3ʹ and the above sequencing kit. DNA sequences were determined using an ABI-PRISM 3100 automatic sequencer (Applied Biosystems).

### SDS-PAGE and western blotting

GM03813 fibroblasts and GM24468-derived MNs were seeded at 1.5 × 10^4^ and 4 × 10^4 ^cells/well in 96-well plates and were treated with TEC-1 or SMN-C3 for 72 h at the concentration of 0.01, 0.03, 0.1, 0.3, 1, or 3 µM. After 72 h exposure of compound, GM24468 derived MNs and GM03813 fibroblasts were washed once with D-PBS (-), lysed with RIPA buffer (Wako) containing cOmplete (Sigma-Aldrich) and PhosSTOP (Sigma-Aldrich). The lysates were spun at 15,000 rpm for 15 min at 4 °C, and the supernatants were collected. The protein concentration was quantified with BCA kit (Wako). The lysates, adjusted to have the same protein amount, were mixed with NuPAGE LDS sample buffer containing DTT and heated at 95 °C for 5 min. Except for HTT detection, each sample (2 µg total protein/lane) was loaded Perfect NT Gel 7.5–15% gel (DRC, Japan) and run at 150 V for 60–70 min with Tris/glycine/SDS running buffer (BIO-RAD, USA). The proteins were transferred from the gel to Immobilon-P PVDF Membrane (Millipore, USA) with a transfer solution comprising 10% methanol and 1 × Novex Tris-glycine transfer buffer at 25 V for 180–240 min using Criterion blotter (BIO-RAD). In the case of HTT detection, each sample (2 µg total protein/lane) was loaded to Perfect NT Gel 7.5–15% gel and run at 150 V for 60–70 min with NuPAGE Tris–Acetate SDS Running Buffer. The proteins were transferred with a transfer solution comprising 10% methanol and 1 × Novex Tris-glycine transfer buffer at 25 V for 960 min using Criterion blotter. The membranes were blocked with Pierce Protein free T20 blocking reagent for 1 h and subsequently incubated with primary antibodies diluted with Can Get Signal Solution 1 (Toyobo) at room temperature for 1 h. Anti-SMN monoclonal antibody (MoAb)^15^ (1:1000 [fibroblasts], 1:2000 [iPSC]) (Cat. No. BD610646, BD Biosciences, USA), GAPDH MoAb^44^ (1:2000, Cat. No. 016-25523, Wako), anti HTT MoAb^45^ (1:1000, Cat. No. MAB2166, Millipore), or Choline acetyltransferase (ChAT) polyclonal antibody^46^ (1:2000, Cat. No. AB144P, Millipore) were used as the primary antibody. The membranes were washed with Tris-buffered Saline with Tween20 (TBS-T) three times and incubated with the secondary antibody, HPR-labeled anti-mouse IgG antibody (1:5000, Cat. No. NA9310-1ML, GE Healthcare, USA) or HRP-labeled anti-Goat IgG antibody (1:5000, Jackson laboratory, USA) diluted with Can Get Signal Solution 2 (Toyobo) at room temperature for 1 h. After washing with TBS-T four times, chemiluminescence signals were detected by Immunostar Zeta (Wako) and LAS-4000 (GE healthcare).

### SMN HTRF

GM24468-derived MNs were seeded at 4 × 10^4^ cells/well in 96-well plates and were treated with TEC-1 for 72 h at the final concentration of 0.03, 0.1, 0.3, 1, and 3 µM.

SMN protein concentration of the cells were quantified using an SMN HTRF kit^15^ (Cat. No. 63ADK033PEG, Cisbio, USA) according to manufacturer’s instructions. The fluorescence emission was read at two different wavelengths (665 nm and 620 nm or 615 nm) using an Envision plate reader (PerkinElmer, USA) excited by 320 nm. The concentration of protein in each cell lysate were measured by BCA kit (BIO-RAD) and used for normalization.

### GALC-enzyme activity assay

GALC enzymatic activities were quantified as reported by Wiederschain et al.^47^. Hs683 cells were seeded at 1.5 × 10^5^ cells/well in 6-well plate. Cells were treated with TEC-1 (0.1, 0.3, or 1 µM) or SMN-C3 (0.03, 0.1, 0.3, or 1 µM) for 3 days. Hs683 cells were washed with D-PBS (–), collected with ultra-pure water using a cell scraper, and homogenized with homogenizers (NS-4, NS310E, Micotechnition, Japan) for 10 s. Cell lysates were centrifuged at 600 × *g* for 10 min at 4 °C, followed by collection of supernatants. The concentration of protein of each sample was quantified with a BCA kit. The samples were adjusted to 1 µg protein/µL (total volume 20 µL) with ultra-pure water and then 80 µL of reaction buffer (0.25% taurocholate, 0.05% oleic acid and 0.1 mol/L citrate, pH 4.4) containing 80 µM 6-Hexadecanoylamino-4-methylumbelliferone b-D-galactopyranoside (HMU-Gal, Carbosynth, UK) was added. Stop buffer (0.2 mol/L Glycine solution with NaOH, pH 10.6) was mixed with an equal amount of ethanol. The mixture was reacted at 37 °C for 20 h. The enzymatic reaction (10 µL) was quenched with 200 µL of stop buffer containing ethanol (EtOH). Signals were detected by reading the fluorescence emission at 450 nm with plate reader (Enspire, PerkinElmer) excited by 385 nm. The fluorescence intensity was corrected by the value of standard substrate of 6-hexadecanoylamino-4-methylumbelliferone (Cat. No. EH10520, Carbosynth).

### Mass spectrometry for determination of psychosine and sphingosine

Hs683 cells were seeded at 1.5 × 10^5^ cells/well in 6-well plates and were treated with TEC-1 or SMN-C3 for 12 days. The sample was extracted with EtOH and dried under nitrogen spray. Pellet was completely re-dissolved in EtOH using vortex and ultrasonication. The resulting sample was centrifuged at 15,000 rpm for 5 min at 4 °C. Analytes were separated from the supernatant and quantified by LC/MS/MS as reported by Boutin et al.^48^.

### Evaluation of micronucleus test

Mitomycin (MMC), cyclophosphamide (CP) and colcemid (COL) were used as positive controls. These articles have been recommended for use in the OECD test guideline (Test No. 487)^49^. For metabolic activation, pre-cultured TK6 cells with the culture medium were supplemented with Sprague–Dawley rat liver mix (S9 mix, IEDA TRADING CORPORATION, Japan) at 3.33 vol%. Then, compound was mixed with the cell suspension and treated under the following treatment conditions:

1. Short-term treatment for 3 h without S9 mix, followed by a 21-h culture (−S9 mix assay),
2. Short-term treatment for 3 h with S9 mix, followed by a 21-h culture (+S9 mix assay), or
3. Continuous treatment for 24 h without S9 mix (24-h assay).

For treatment conditions 1 and 2, the cells in each well were centrifuged after the 3-h treatment, the supernatants were discarded, and the cells were washed twice with culture medium. The cells were further cultured with fresh culture medium for 21 h. The compound-treated cells were examined by the unaided eye for precipitation of compound at the start and end of treatment. After treatment, the rest of each cell suspension was centrifuged and then the cells were suspended in a 0.075 mol/L KCl solution at room temperature for 10 min. Then, 25 vol% acetic acid containing methanol was added and the centrifuged cells were fixed. The fixed cells were resuspended in a small amount of 3 vol% acetic acid containing methanol, which was changed two times by centrifugation. Finally, a drop of the cell suspension was transferred onto a slide and dried at room temperature. The slides were stained at room temperature in a 3 vol% Giemsa solution (Merck KGaA) diluted with Sörensen phosphate buffer (1/15 mol/L, pH 6.8, Muto Pure Chemicals Co. Ltd.). Four slides per dose (2 slides per well) were prepared. Each slide was coded randomly. The test was conducted at the following doses of TEC-1. -S9 mix assay: 1.5, 1.7, 1.9, 2.1, 2.3, 2.5, 2.7, 2.9, and 3.1 µg/mL, + S9 mix assay: 1.9, 2.1, 2.3, 2.5, 2.7, 2.9, 3.1, 3.3, and 3.5 µg/mL, 24-h assay: 0.9, 1.1, 1.3, 1.5, 1.7, 1.9, 2.1, 2.3, and 2.5 µg/mL. To perform scoring for compound and negative control groups, the number of cells in the suspensions was counted using a Coulter Counter (Z2; Beckman Coulter, Inc.). The positive control groups were not determined. A portion (0.5 mL) of the cell suspension was diluted with 20 mL of Isoton II, and 0.5 mL of this solution was used for cell counts. The cell number per 1 mL at each culture was calculated by the following formula.

$${\text{Cell}}\,{\text{number}}\,\left( {/{\text{mL}}} \right)\, = \,{\text{Dilution}}\,{\text{factor}}\, \times \,\left[ {\left( {{\text{value}}\,{\text{of}}\,1{\text{st}}\,{\text{count}}\, + \,{\text{value}}\,{\text{of}}\,2{\text{nd}}\,{\text{count}}} \right)/2 \times 2} \right]$$

The population doubling (PD) and relative population doubling (RPD) at each concentration was calculated by the following formula.

$$\begin{aligned} {\text{PD}} & { = }\left[ {{\text{log1}}0 \, \left( {{\text{cell number at the end of culture }}/{\text{ cell number at the start of treatment}}*} \right)} \right] \, /{\log}_{{{1}0}} {2} \\ & *:{ 1}\, \times \,{1}0^{{5}} {\text{cells}}/{\text{mL}}. \\ \end{aligned}$$

$${\text{RPD }}\left( \% \right) \, = \, \left[ {\left( {{\text{PD of the compound}}{-}{\text{treated group}}} \right) \, / \, \left( {\text{PD of the negative control group}} \right)} \right] \, \times 100$$

For the treated groups, the 3 consecutive concentrations were selected as follows. For −S9 mix assay condition, cytotoxicity, as indicated by a RPD of less than 50% was noted at 2.3 μg/mL of TEC-1 and higher; for + S9 mix assay condition, cytotoxicity was noted at 2.9 μg/mL of TEC-1 and higher; for 24-h assay condition, precipitation was noted at 1.5 μg/mL of TEC-1 and higher. Based on these results 1.9, 2.1 and 2.3 μg/mL of TEC-1 were analyzed for −S9 mix assay condition; 2.5, 2.7, and 2.9 μg/mL of TEC-1 were analyzed for + S9 mix assay condition; 1.1, 1.3, 1.5, μg/mL of TEC-1 were analyzed for 24-h assay condition. For all concentrations, 4000 mononuclear cells with cytoplasm were analyzed and the number of cells with micronucleus (micronuclei) was counted using a microscope (× 600). Micronucleus is identified as a round or oval small nucleus in the cytoplasm with the same staining intensity as the main nucleus. The diameter of each micronucleus should be less than 1/2 of the diameter of the main nucleus. Two investigators observed the slides. Each investigator scored 1000 cells for the frequency of micronucleated cells. Data from the two investigators were combined for each culture and the combined data from duplicate cultures were pooled for each concentration for each treatment condition. A cell having one or more micronuclei was recorded as a micronucleated cell. The test article was judged to be positive if the incidence of micronucleated cells between any test article treatment groups satisfied both criteria (1) and (2) shown below.

1. A significant difference in the incidence of micronucleated cells between the negative control group is detected in the statistical analysis and concentration-dependent increase was also detected in the statistical analysis.
2. The incidence of micronucleated cells is more than the historical control range (mean + 2SD).

Otherwise, the test article was judged to be negative.

### Pharmacokinetic study in adult wild type FVB mice

TEC-1 was administered at doses of 2, 6, and 20 mg/kg for oral (suspended in 0.5% methyl cellulose aqueous solution) to male FVB mice (aged 8 weeks, 3 mice per a time point). After 0.5–120 h administration, blood and brain samples were collected. Blood samples with heparin were centrifuged for plasma collection. Brain samples were homogenized in saline 20% (w/v). The samples were deproteinized with acetonitrile and following by centrifugation, and the supernatants were analyzed by LC/MS/MS to obtain TEC-1 concentrations of the plasma and brain.

### Pharmacokinetic study in neonatal FVB mice

TEC-1 was intraperitoneally administered at doses of 0.2, 0.6, and 2 mg/kg (suspended in saline/0.9% NaCl solution) to male FVB mice (10 days old, 3 mice per a time point). After administration, brain samples and blood samples with heparinized capillary tubes from heart were collected. Blood samples were centrifuged to obtain the plasma fraction. Brain samples were homogenized in 20% (w/v) saline. The samples were deproteinized with acetonitrile followed by centrifugation and the supernatants were analyzed by LC/MS/MS to obtain TEC-1 concentrations of the plasma and brain.

### Animals and in vivo studies

A pair of heterozygous SMNΔ7 mice^28^ (mSmn+/−, SMN2+/+, SMNΔ7+/+) were purchased from Jackson Laboratory (USA, stock number 005025). The homozygous SMNΔ7 mice (affected and referred to as “SMNΔ7 mice”; mSmn−/−, SMN2+/+, SMNΔ7+/+) was generated by cross-bleeding of heterozygous SMNΔ7 mice. All experiments were approved and monitored by the animal experiment committee of Gifu Pharmaceutical University and were performed after approval by the Bioethics and Biosafety Committee of Gifu Pharmaceutical University. All procedures relating to animal care and treatment confirmed to animal care guidelines issued by the National Institutes of Health.

### Administration of TEC-1

SMNΔ7 mice were treated with 2, 6, and 20 mg/kg TEC-1 (succinate) once a day by intraperitoneal injection (TERUMO, Japan) from P2 to P23. TEC-1 was diluted in 0.9% NaCl (aqueous) for intraperitoneal injection. After P24, TEC-1 was administrated one a day orally using 0.4 × 0.7 mm feeding needle (Primetech Corporation, Japan). For oral administration, TEC-1 was diluted in distilled water (Otsuka Pharmaceutical Factory, Japan). Heterozygous mice received equal volumes of vehicle.

### Behavioral analysis

Survival and body weight were assessed daily. Righting reflex test^15^ tests were performed daily at P6, 11 and 16. Righting time was defined as the time it took a pup to turn over to its front after being placed completely on its back (maximum 60 s). Dead individuals were set to 60 s.

### Statistical analysis

No statistical methods were used to predetermine sample sizes, but the sample sizes we used were consistent with those generally employed in the field. Comparisons between two groups were analyzed by Student’s t-test (unpaired, two-tailed). Dose-responsibility test was analyzed by one-way analysis of variance followed by William’s test or Dunnett’s test. In micronucleus test, Fisher’s exact test was performed in order to compare the incidence of micronucleated cells in the test article groups or the positive control group with that of the negative control groups for each treatment condition. When Fisher’s exact test showed statistical significance, the exact Cochran-Armitage trend test was performed to evaluate any dose–response relationship. Statistical processing was performed using the Microsoft Excel or GraphPad Prism Software (Prism 6 or 8), EXSUS statistical software package (CAC Croit Corporation, Japan).

## Supplementary information

Supplementary Information.

## Data Availability

The datasets generated and/or analyzed during the current study are available from the corresponding author upon reasonable request.
