# Supplementary material for: Discovery of a CNS penetrant small molecule SMN2 splicing modulator with improved tolerability for spinal muscular atrophy
Source: Sci Rep. 2020 Oct 15;10:17472. doi: 10.1038/s41598-020-74346-9 (PMC7562719; doi:10.1038/s41598-020-74346-9)
Supplement: Supplementary file 1 — Supplementary Information. [file 41598_2020_74346_MOESM1_ESM.docx]

**Supplementary information**

**Discovery of a CNS penetrant small molecule SMN2 splicing modulator with improved tolerability for spinal muscular atrophy**

Shiori Ando^1, #^, Shunya Suzuki^2, #^, Shoichi Okubo^2^, Kazuki Ohuchi^1^, Kei Takahashi^1^, Shinsuke Nakamura^1^, Masamitsu Shimazawa^1^, Koji Fuji^2^ & Hideaki Hara^1, *^

^1^ Molecular Pharmacology, Department of Biofunctional Evaluation, Gifu Pharmaceutical University, Gifu, 501-1196, Japan.

^2^ Reborna Biosciences Inc., Kanagawa, 251-0012, Japan.

^#^ Shiori Ando and Shunya Suzuki contributed equally to this work.

*Address correspondence to:

Hideaki Hara, Ph.D., R.Ph.

Molecular Pharmacology, Department of Biofunctional Evaluation, Gifu Pharmaceutical University, 1-25-4 Daigaku-nishi, Gifu, 501-1196, Japan**.**

Tel and Fax :+81-58-230-8126

E-mail: **hidehara@gifu-pu.ac.jp**

**a**

**Supplementary Figure 1.** **Risdiplam reduced *FOXM1b/c* in cynomolgus monkey fibroblasts.**

**a** Schematic diagram and nucleotide sequences of the human and cynomolgus monkey *FOXM1* genomes around cryptic exons. Sequence lengths (nt) are shown (above or below). Exon 9 is indicated with colored boxes and exon regions included by *ΔC* isoforms are indicated with red boxes. Exon 9 sequences are shown in uppercase, and introns are shown in lowercase. The nucleotides highlighted in yellow indicate different positions between cynomolgus monkeys and humans. **b**–**d** Effects of TEC-1 and risdiplam on *FOXM1* transcripts (*1b/c*, *1a*, *ΔC*) of cynomolgus monkey fibroblasts. Cynomolgus monkey fibroblasts (NCMDF) were exposed to compounds for 24 h. qPCR was performed with primers and the probe of *FOXM1* with common sequences of humans and monkeys, normalized by *GAPDH* with the delta CT method. Data in **b**, **c**, **d** represent means ± SEM of 4 or 6 independent assessments per concentration. **e** RT-PCR analysis of *FOXM1* variants of GM03813 exposed for 24 h to the compound. RT-PCR products of *FOXM1* variants were separated with agarose gel electrophoresis and visualized with ethidium bromide. Standard *FOXM1* fragments corresponding to the three isoforms (Oligo STD) are shown. Molecular weight markers are shown on the right (bp).

**e**

**d**

**c**

**b**

**b**

**a**

**c**

**Supplementary Figure 2. Cryptic exon inclusion in *GALC* mRNA by SMN-C3.**

**a** Detection of *GALC* mRNA of human oligodendroglioma (Hs683) cells. Hs683 cells were exposed to the compounds (1 μM TEC-1 or 1 μM SMN-C3) for 6 h and with or without CHX. RT-PCR products of *GALC* were separated with agarose gel electrophoresis and visualized with ethidium bromide. **b** Electropherogram of *GALC* sequencing. The RT-PCR fragment shown in **a** (SMN-C3 and CHX treated) was analyzed. After the red vertical line, the waveform data of exon 7 (main peak) overlapped with those of the cryptic exon sequence (minor peak). **c** RT-PCR analysis of *GALC* transcripts of GM03813 cells treated for 6 h with the compounds and CHX. RT-PCR products of *GALC* were separated with agarose gel electrophoresis and visualized with ethidium bromide (left). Standard *GALC* fragments with or without the cryptic exon (Oligo STD) are shown. RT-PCR products of *GAPDH* were separated with agarose gel electrophoresis and visualized with ethidium bromide (right). Standard GAPDH (Oligo STD) is shown. Molecular weight markers are shown on the right (bp).

**a**

**Supplementary Figure 3. The SMN-C series, including risdiplam, reduced total *GALC* mRNA levels in monkey but not dog and rat cells.**

**a** Schematic diagram and nucleotide sequences of the *GALC* genome around the cryptic exon. Nucleotide lengths are shown (below). The included exon by the SMN-C series is shown in uppercase, and introns are shown in lowercase. In-frame stop codons in cryptic exons and different nucleotides of simian sequences from human sequences are highlighted in green and yellow, respectively. **b**–**e** *GALC* mRNA reduction by each compound in indicated cells. qPCR analysis of *GALC* transcripts of each cell type treated for 24 h with the compounds. Data were normalized by *GAPDH* with the delta CT method, and are represented as means ± SEM of 4 or 8 independent assessments per concentration.

**e**

**d**

**c**

**b**

**Supplementary Figure 4. Cryptic exon inclusion and reduced expression of HTT by SMN-C3.**

**a** Western blots of GM03813 treated with the compound for 3 days. Each protein and its antibody for detection is indicated. Molecular weight markers are shown on the left (kDa). The amounts of full-length HTT protein, normalized with that of GAPDH protein, used for dose-response curves in Fig. 4e. **b** Electropherogram of *HTT* sequencing. PCR products of Hs683 cells exposed for 6 h to both risdiplam and CHX were sequenced directly with a genetic analyzer. After the red vertical line, the waveform data of exon 50 sequence (main peak) are overlapped by those of the cryptic exon (minor peak).

**a**

**Supplementary Figure 5. TEC-1 did not substantially impact the inclusion of the *HTT* cryptic exon in human.**

**a** Schematic diagram and nucleotide sequences of the human *HTT* genomes around cryptic exons. Nucleotide lengths are shown. The cryptic exon is indicated by a red box. The cryptic exon sequence included by risdiplam is shown in uppercase letters, introns are shown in lowercase letters. In-frame stop codon in cryptic exon is highlighted in green. **b** RT-PCR analysis of *HTT* transcripts of GM03813 treated for 6 h with compounds and CHX. RT-PCR products of HTT were separated with agarose gel electrophoresis and visualized with ethidium bromide (left). Standard *HTT* fragments with or without the cryptic exon (Oligo STD) are shown. RT-PCR products of GAPDH were separated with agarose gel electrophoresis and visualized with ethidium bromide (right). Standard GAPDH (Oligo STD) is shown. Molecular weight markers are shown on the right (bp).

**b**

**a**

**b**

**Supplementary Figure 6. SMN-C3 also modulated *SMN* splicing and increased SMN protein in the motor neurons derived from SMA patient iPSCs (GM24468).**

**a** qPCR analysis of the motor neurons derived from SMA patient iPSCs. The motor neurons were exposed to SMN-C3 for 24 h. The amounts of mRNA, including (FL-*SMN2*), and excluding (Δ7) exon 7, were normalized with those of *GAPDH*. **b** SMN protein levels of motor neurons treated with SMN-C3 for 3 days. SMN protein in the cell lysate was quantified with HTRF and normalized with total protein. Data in **a**, **b** represent means ± SEM of three independent assessments per concentration. p < 0.05 *, p < 0.01 **, p < 0.001 *** as assessed by one-way ANOVA followed by Dunnett’s test using DMSO-treated cells as a control.

**b**

**a**

**Supplementary Figure 7**. **TEC-1 did not inhibit the significant therapeutic effect of nusinersen in SMA patient-derived fibroblasts.**

qPCR of SMN transcripts of GM03813 cells exposed to TEC-1 and nusinersen (100 nM), which was prepared with Lipofectamine 2000, for 24 h. The amount of FL-*SMN2* (**a**) and Δ7 (**b**) mRNA was quantified with qPCR and normalized with that of *GAPDH*. Data represent means ± SEM of four independent assessments per concentration. p < 0.01 **, p < 0.001 *** as assessed by Student’s t-test (two-tailed) using DMSO-treated cells as a control. ^#^<0.025 as assessed by William’s test (one-tailed) using nusinersen alone-treated cells as a control.

**b**

**a**

| **Supplementary Table 4**. Pharmacokinetic parameters of TEC-1 after oral single administrations in adult FVB mice | | | | | | | | | | | |
| --- | --- | --- | --- | --- | --- | --- | --- | --- | --- | --- | --- |
|  | **Plasma** | | | | |  | **Brain** | | | | |
| **Dose** | **2 mg/kg** |  | **6 mg/kg** |  | **20 mg/kg** |  | **2 mg/kg** |  | **6 mg/kg** |  | **20 mg/kg** |
|  | **Mean** |  | **Mean** |  | **Mean** |  | **Mean** |  | **Mean** |  | **Mean** |
| **C_max_ (ng/mL or ng/g)** | **136.4** |  | **157.2** |  | **222.0** |  | **167.8** |  | **195.4** |  | **360.4** |
| **T_max_ (h)** | **3.00** |  | **3.00** |  | **16.00** |  | **1.00** |  | **7.00** |  | **32.00** |
| **AUC_0–24 h_ (ng･h/mL or ng･h/g)** | **1906.7** |  | **3100.3** |  | **4950.5** |  | **2234.1** |  | **3956.7** |  | **6322.1** |
| **AUC_0–120 h_ (ng･h/mL or ng･h/g)** | **2095.9** |  | **6647.9** |  | **16350.5** |  | **2433.7** |  | **8011.1** |  | **29429.3** |
| **MRT (h)** | **10.71** |  | **33.14** |  | **48.46** |  | **10.67** |  | **29.54** |  | **54.58** |
| **Kp (AUC_0–120h_ ratio)** | **-** |  | **-** |  | **-** |  | **1.2** |  | **1.2** |  | **1.8** |
| **(n = 3)** |  |  |  |  |  |  |  |  |  |  |  |

**Supplementary Figure 8. Concentration curve of TEC-1 in the plasma and brain of adult mice.**

Concentration curve of TEC-1 in single shot administered orally (p.o.) (8-week-old FVB mice. Doses: 2, 6, 20 mg/kg). Concentration was determined by LC/MS/MS at several time points after administration. Data represent means ± standard deviation (SD) of three independent assessments per concentration.

**b**

**a**

| **Supplementary Table 5.** Pharmacokinetic parameters of TEC-1 after intraperitneal single administration in juvenile FVB mice | | | | | | | | | | | |
| --- | --- | --- | --- | --- | --- | --- | --- | --- | --- | --- | --- |
|  | Plasma | | | | |  | Brain | | | | |
| **Dose** | **0.2 mg/kg** |  | **0.6 mg/kg** |  | **2 mg/kg** |  | **0.2 mg/kg** |  | **0.6 mg/kg** |  | **2 mg/kg** |
|  | **Mean** |  | **Mean** |  | **Mean** |  | **Mean** |  | **Mean** |  | **Mean** |
| **C_max_ (ng/mL or ng/g)** | **2.2** |  | **11.3** |  | **42.0** |  | **10.5** |  | **63.2** |  | **218.9** |
| **T_max_ (h)** | **0.50** |  | **0.50** |  | **3.00** |  | **0.50** |  | **1.00** |  | **1.00** |
| **AUC_0–24 h_ (ng･h/mL or ng･h/g)** | **15.2** |  | **129.9** |  | **718.6** |  | **69.7** |  | **600.3** |  | **3547.9** |
| **AUC_0–120 h_ (ng･h/mL or ng･h/g)** | **15.2** |  | **149.1** |  | **1829.8** |  | **87.7** |  | **716.7** |  | **8261.5** |
| **MRT (h)** | **7.21** |  | **10.74** |  | **40.37** |  | **11.19** |  | **11.78** |  | **35.78** |
| **Kp (AUC_0–120h_ ratio)** | **-** |  | **-** |  | **-** |  | **5.8** |  | **4.8** |  | **4.5** |
| **(n = 3, pool)** |  |  |  |  |  |  |  |  |  |  |  |

**Supplementary Figure 9. Concentration curve of TEC-1 in the plasma and brain of juvenile FVB mice**.

Concentration curve of TEC-1 in single dose administered intraperitoneally (i.p.) (10-day-old FVB mice. Dose: 0.2, 0.6, 2 mg/kg). Concentration was determined by LC/MS/MS at several time points after administration. Data represent means of one point (pooled for three mice) per concentration.

**Supplementary Figure 10**. **TEC-1 increased SMN and ChAT protein levels in the motor neurons derived from SMA patient iPSCs (GM24468).**

Protein level of motor neurons derived from SMA patient iPSCs treated with the compound for 3 days. Following western blot analyses, the amounts of SMN and ChAT proteins signal were analyzed and normalized with those of the GAPDH protein signal. The same cell lysate was analyzed with each antibody indicated (SMN or ChAT, GAPDH). Molecular weight markers are shown on the left (kDa). The normalized data are shown in Fig. 5c, d.

**Supplementary Figure 11**. **Alignment of the included (cryptic) exon sequences as a result of the SMN-C series, and proceeding scenarios expected in animals.**

The (cryptic) exon regions are shown in uppercase, and intron regions are shown in lowercase. Nucleotides that differ between the *SMN1* and *SMN2* genes are highlighted in green. Each nucleotide highlighted in yellow indicates a different position between human *FOMX1* exon 9/A2 and the human *GALC* cryptic exon.

**Supplemental Table 1**. Primers and probes or TaqMans for qPCR

| Detection | Species | Type | Name | Sequence |
| --- | --- | --- | --- | --- |
| FL-SMN | Human | Primer | 003‐Fw‐FL‐SMN | 5’－GCTCACATTCCTTAAATTAAGGAGAAA－3’ |
|  |  | Primer | 005－Rv－SMN | 5’－TCCAGATCTGTCTGATCGTTTCTT－3’ |
|  |  | Probe | 004－Probe－SMN | 5’-CTGGCATAGAGCAGCACTAAATGACACCAC-3’ |
| Δ7 | Human | Primer | 006－Fw－delta7 | 5’－TGGCTATCATACTGGCTATTATATGGAA－3’ |
|  |  | Primer | 005－Rv－SMN |  |
|  |  | Probe | 004－Probe－SMN |  |
| Cryptic GALC | Human | Primer | 43-Fw-GALC (Exon 5) | 5'-TATTATGTCGTGACCTGGATTGT-3’ |
|  |  | Primer | 46-Rv-GALC (cryptic exon) | 5'-AACCAAATACCACCTATTCTCCA-3’ |
|  |  | Probe | 44-Probe-GALC (Exon 5) | 5'- CGCCAAGCGTTACCATGATTTGGAC-3’ |
| GALC | Human | Taqman | Hs01012300_m1 |  |
| GALC | Monkey | Taqman | Rh01012291_m1 |  |
| GALC | Dog | Taqman | Cf02627998_m2 |  |
| GALC | Rat | Taqman | Rn01775763_g1 |  |
| GAPDH | Human | Taqman | Hs99999905_m1 |  |
| GAPDH | Monkey | Taqman | Rh02621745_g1 |  |
| GAPDH | Dog | Taqman | Cf04419463_gH |  |
| GAPDH | Rat | Taqman | Rn01517759_m1 |  |
| Cryptic HTT | Human & monkey | Primer | 62-Fw-HTT-Exon 49 | 5'-TCTCCAAACTGCCCAGTCA-3’ |
|  |  | Primer | 64-Rv-HTT-Exon 49B | 5'- ATCTCTCCTGGGCTACGTG-3’ |
|  |  | Probe | 63-Probe-HTT-Exon 49 | 5'-GCACCTTCCTCCTGAGAAAGAGAAGG-3’ |
| HTT | Human & monkey | Primer | 62-Fw-HTT-Exon 49 |  |
|  |  | Primer | 65-Rv-HTT-Exon 49-50 | 5'-CCAGGACAGGGCCTCAA-3’ |
|  |  | Probe | 63-Probe-HTT-Exon 49 |  |
| FOXM1a | Human & monkey | Primer | 54-Fw-FOXM1 (Exon 8) | 5'-GCTTCCCTCATGAGCTCAGA-3’ |
|  |  | Primer | 5a-Rv-FOXM1 (Exon 9)_h&m | 5'-CAGATCGCCACTAAAGAACTTACT-3’ |
|  |  | Probe | 55-Probe-FOXM1-Exon 8 | 5'-CCATAGCAAGCGAGTCCGCATTG-3’ |
| FOXM1b/c | Human & monkey | Primer | 54-Fw-FOXM1 (Exon 8) |  |
|  |  | Primer | 57-Rv-FOXM1 (Exon 8-10) | 5'-TCAGCTAGCAGCACCTTGG-3’ |
|  |  | Probe | 55-Probe-FOXM1-Exon 8 | ’ |
| FOXM1c | Human & monkey | Primer | 54-Fw-FOXM1 (Exon 8) |  |
|  |  | Primer | 58-Rv-FOXM1 (cryptic-Exon 10) | 5’-CTCAGCTAGCAGCACTCATG-3’ |
|  |  | Probe | 55-Probe-FOXM1-Exon 8 |  |
|  |  |  |  |  |

**Supplemental Table 2**. Primers and Probes for RT-PCR

| Detection | Type | Name | Sequence |
| --- | --- | --- | --- |
| GALC | Primer | 43-Fw-GALC (Exon 5) | 5'-TATTATGTCGTGACCTGGATTGT-3’ |
|  | Primer | 49-Rv-GALC (Exon 7) | 5’- ACTCGCTGGAGACCTTGATA-3’ |
| HTT | Primer | 62-Fw-HTT-Exon 49 | 5'-TCTCCAAACTGCCCAGTCA-3’ |
|  | Primer | 66-Rv-HTT-Exon 50 | 5’-GGGATCTGCTCATGGATCAAA-3’ |
| FOXM1 | Primer | 54-Fw-FOXM1 (Exon 8) | 5'-GCTTCCCTCATGAGCTCAGA-3’ |
|  | Primer | 59-Rv-FOXM1 (Exon 10) | 5’-CTGCAGAAGAAAGAGGAGCTAT-3’ |
| GAPDH | Taq-man | Hs99999905_m1 | Unpublished |

**Supplemental Table 3**. Sequences of oligo DNA standards.

| Name | Sequence |
| --- | --- |
| Standard FL-SMN | 5’TCAGATAACATCAAGCCCAAATCTGCTCCATGGAACTCTTTTCTCCCTCCACCACCCCCCATGCCAGGGCCAAGACTGGGACCAGGAAAGATAATTCCCCCACCACCTCCCATATGTCCAGATTCTCTTGATGATGCTGATGCTTTGGGAAGTATGTTAATTTCATGGTACATGAGTGGCTATCATACTGGCTATTATATGGGTTTCAGACAAAATCAAAAAGAAGGAAGGTGCTCACATTCCTTAAATTAAGGAGAAATGCTGGCATAGAGCAGCACTAAATGACACCACTAAAGAAACGATCAGACAGATCTGGAATGTGAAGCGTTATAGAAGATAACTGGCCTCATTTCTTCAAAATATCAAGTGTTGGGAAAGAAAAAAGGAAGTGGAATGGGTAACTCTTCTTGATTAAAAGTTATGTAATAACCAAATGCAATGTGAAATATTTTACTGGACTCTATTTTGAAAAACCATCTGTAAAAGACTGGGGTGGGGGT-3’ |
| Standard Δ7 | 5’ACCACCCCCCATGCCAGGGCCAAGACTGGGACCAGGAAAGCCAGGTCTAAAATTCAATGGCCCACCACCGCCACCGCCACCACCACCACCCCACTTACTATCATGCTGGCTGCCTCCATTTCCTTCTGGACCACCAATAATTCCCCCACCACCTCCCATATGTCCAGATTCTCTTGATGATGCTGATGCTTTGGGAAGTATGTTAATTTCATGGTACATGAGTGGCTATCATACTGGCTATTATATGGAAATGCTGGCATAGAGCAGCACTAAATGACACCACTAAAGAAACGATCAGACAGATCTGGAATGTGAAGCGTTATAGAAGATAACTGGCCTCATTTCTTCAAAATATCAAGTGTTGGGAAAGAAAAAAGGAAGTGGAATGGGTAACTCTTCTTGATTAAAAGTTATGTAATAACCAAATGCAATGTGAAATATTTTACTGGACTCTATTTTGAAAAACCATCTGTAAAAGACTGGGGTGGGGGTGGGAGGCC-3’ |
| Standard GAPDH | 5’AAATTGAGCCCGCAGCCTCCCGCTTCGCTCTCTGCTCCTCCTGTTCGACAGTCAGCCGCATCTTCTTTTGCGTCGCCAGCCGAGCCACATCGCTCAGACACCATGGGGAAGGTGAAGGTCGGAGTCAACGGATTTGGTCGTATTGGGCGCCTGGTCACCAGGGCTGCTTTTAACTCTGGTAAAGTGGATATTGTTGCCATCAATGACCCCTTCATTGACCTCAACTACATGGTTTACATGTTCCAATATGATTCCACCCATGGCAAATTCCATGGCACCGTCAAGGCTGAGAACGGGAAGCTTGTCATCAATGGAAATCCCATCACCATCTTCCAGGAGCGAGATCCCTCCAAAATCAAGTGGGGCGATGCTGGCGCTGAGTACGTCGTGGAGTCCACTGGCGTCTTCACCACCATGGAGAAGGCTGGGGCTCATTTGCAGGGGGGAGCCAAAAGGGTCATCATCTCTGCCCCCTCTGCTGATGCCCCCATGTTCGTCAT-3’ |
| Standard human GALC (for Hs01012300_m1) | 5’CGGCACTGAGCCCTCCCACATGCATTATGCACTAGATGAGAATTATTTCCGAGGATACGAGTGGTGGTTGATGAAAGAAGCTAAGAAGAGGAATCCCAATATTACACTCATTGGGTTGCCATGGTCATTCCCTGGATGGCTGGGAAAAGGTTTCGACTGGCCTTATGTCAATCTTCAGCTGACTGCCTATTATGTCGTGACCTGGATTGTGGGCGCCAAGCG-3’ |
| 41-Cryptic exon-GALC-STD | 5’TTCAGCTGACTGCCTATTATGTCGTGACCTGGATTGTGGGCGCCAAGCGTTACCATGATTTGGACATTGATTATATTGGAATTTGGAATGAGAGGTCATATAATGCCAATTATATTAAGGTTTTTGGAGAATAGGTGGTATTTGGTTACATGAATATTAAGAAAAATGCTGAATTATCAAGGTCTCCAGCGAGTGAAAATCATAGCAAGTGATAATCTCTGGGAGTCCATCTCTGCATCCATGCTCCTTGATGCCGAACTCTTCAAGGTGGTTGATGTTATAGG-3’ |
| 51-FOXM1a-E8-9-10-STD | 5’GGCGGAAGATGAAGCCACTGCTACCACGGGTCAGCTCATACCTGGTACCTATCCAGTTCCCGGTGAACCAGTCACTGGTGTTGCAGCCCTCGGTGAAGGTGCCATTGCCCCTGGCGGCTTCCCTCATGAGCTCAGAGCTTGCCCGCCATAGCAAGCGAGTCCGCATTGCCCCCAAGGTTTTTGGGGAACAGGTGGTGTTTGGTTACATGAGTAAGTTCTTTAGTGGCGATCTGCGAGATTTTGGTACACCCATCACCAGCTTGTTTAATTTTATCTTTCTTTGTTTATCAGTGCTGCTAGCTGAGGAGGGGATAGCTCCTCTTTCTTCTGCAGGACCAG-3’ |
| 52-FOXM1bc-ΔA2-STD | 5’GGCGGAAGATGAAGCCACTGCTACCACGGGTCAGCTCATACCTGGTACCTATCCAGTTCCCGGTGAACCAGTCACTGGTGTTGCAGCCCTCGGTGAAGGTGCCATTGCCCCTGGCGGCTTCCCTCATGAGCTCAGAGCTTGCCCGCCATAGCAAGCGAGTCCGCATTGCCCCCAAGGTGCTGCTAGCTGAGGAGGGGATAGCTCCTCTTTCTTCTGCAGGACCAG-3’ |
| 53-FOXM1c-Cryptic exon-STD | 5’GGCGGAAGATGAAGCCACTGCTACCACGGGTCAGCTCATACCTGGTACCTATCCAGTTCCCGGTGAACCAGTCACTGGTGTTGCAGCCCTCGGTGAAGGTGCCATTGCCCCTGGCGGCTTCCCTCATGAGCTCAGAGCTTGCCCGCCATAGCAAGCGAGTCCGCATTGCCCCCAAGGTTTTTGGGGAACAGGTGGTGTTTGGTTACATGAGTGCTGCTAGCTGAGGAGGGGATAGCTCCTCTTTCTTCTGCAGGACCAG-3’ |
| 60-HTT-E49-49B-50-STD | 5’GGGATGCTGCACTGTATCAGTCCCTGCCCACTCTGGCCCGGGCCCTGGCACAGTACCTGGTGGTGGTCTCCAAACTGCCCAGTCATTTGCACCTTCCTCCTGAGAAAGAGAAGGACATTGTGAAATTCGTGGTGGCAACCCTTGAGAGGCAAGCCCTGGTGCTGTGGGAGCCCCAAGGAAGAGCCTCTGGCCTGGTGGCCACGTAGCCCAGGAGAGATTTCTACAGGAGCCCACAGCGCTGAAGGAGAGAGAGGCAGCAGAGCCCTGTCCTGGCATTTGATCCATGAGCAGATCCCGCTGAG-3’ |
| 61-HTT-E49-50-STD | 5’GGGATGCTGCACTGTATCAGTCCCTGCCCACTCTGGCCCGGGCCCTGGCACAGTACCTGGTGGTGGTCTCCAAACTGCCCAGTCATTTGCACCTTCCTCCTGAGAAAGAGAAGGACATTGTGAAATTCGTGGTGGCAACCCTTGAGGCCCTGTCCTGGCATTTGATCCATGAGCAGATCCCGCTGAGTCTGGATCTCCAGGCAGGGCTGGACTGCTGCTGCCTGGCCCTGCAGCTGCCTGGCCT-3’ |
